# New developments in anti-malarial target candidate and product profiles

**DOI:** 10.1186/s12936-016-1675-x

**Published:** 2017-01-13

**Authors:** Jeremy N. Burrows, Stephan Duparc, Winston E. Gutteridge, Rob Hooft van Huijsduijnen, Wiweka Kaszubska, Fiona Macintyre, Sébastien Mazzuri, Jörg J. Möhrle, Timothy N. C. Wells

**Affiliations:** 1 Medicines for Malaria Venture, Route de Pré Bois 20, 1215 Geneva 15, Switzerland; 2 Neglected Infectious Diseases Consulting, Sevenoaks, Kent UK; 3 FSG, Rue de Chantepoulet 25, 1201 Geneva, Switzerland

**Keywords:** Malaria, *Plasmodium*, Elimination drug discovery, Eradication drug discovery, Medicines, Target candidate profile, Target product profile

## Abstract

A decade of discovery and development of new anti-malarial medicines has led to a renewed focus on malaria elimination and eradication. Changes in the way new anti-malarial drugs are discovered and developed have led to a dramatic increase in the number and diversity of new molecules presently in pre-clinical and early clinical development. The twin challenges faced can be summarized by multi-drug resistant malaria from the Greater Mekong Sub-region, and the need to provide simplified medicines. This review lists changes in anti-malarial target candidate and target product profiles over the last 4 years. As well as new medicines to treat disease and prevent transmission, there has been increased focus on the longer term goal of finding new medicines for chemoprotection, potentially with long-acting molecules, or parenteral formulations. Other gaps in the malaria armamentarium, such as drugs to treat severe malaria and endectocides (that kill mosquitoes which feed on people who have taken the drug), are defined here. Ultimately the elimination of malaria requires medicines that are safe and well-tolerated to be used in vulnerable populations: in pregnancy, especially the first trimester, and in those suffering from malnutrition or co-infection with other pathogens. These updates reflect the maturing of an understanding of the key challenges in producing the next generation of medicines to control, eliminate and ultimately eradicate malaria.

## Background

Nearly a decade has passed since the announcement by the World Health Organization (WHO) and the Bill & Melinda Gates Foundation of the long-term goal of eradication of malaria. Use of existing therapy over that period has helped drive a profound decrease in mortality and morbidity of 60 and 37% compared to that at the start of the Millennium [1]. Clear ‘road maps’ on the types of new medicines have now been discussed by the malaria community [2], and an ambitious goal of a further 90% reduction in morbidity and mortality set by the WHO [3]. Four years ago, a proposal was published for the types of molecules [target candidate profiles (TCP)] and medicines (target product profiles (TPP) needed [4], setting clear goals for new therapy. In this document, these concepts are updated, reflecting what has been learned about new medicines in the ‘pipeline’, what has been learned about the challenges of elimination and eradication, and the changing landscape of malaria. The ‘candidate’ in TCP refers to an individual molecule, and in the Medicines for Malaria Venture (MMV) portfolio, these compounds are in formal regulatory preclinical safety assessment or human volunteer studies. The ‘product’ in TPP refers to a final product, which may contain two or more active candidates, and, importantly, the appropriate formulation.

TPPs are strategic tools used to provide guidance during drug discovery and development. While not mandatory, the US Food and Drug Administration (FDA) has published draft TPP guidelines [5], as these documents facilitate communication with regulators. As a minimum, TPPs provide platforms for a shared agreement about what constitutes success. In public healthcare, the publication and feedback for TPPs is a critical part of their refinement. Since drug development takes over a decade to complete, TCPs and TPPs are living documents; they need to be updated to reflect changes in patient needs and the clinical landscape, new safety findings and technical progress (Table 1).

**Table 1:** Overview of newly defined TPPs and TCPs

Profile	Intended use
TPP-1	Case management; treatment of acute uncomplicated malaria in children or adults. Uses a combination of two or more molecules with TCP-1 activity, plus TCP-5 for reducing transmission and TCP-3 for relapse prevention, when such molecules become available For severe malaria, a parenteral formulation of a single fast-acting TCP-1 would be appropriate
TPP-2	Chemoprotection: given to subjects migrating into areas of high endemicity, or during epidemics. Uses a combination of TCP-4 activity, potentially with TCP-1 support for emerging infections
TCP-1	Molecules that clear asexual blood-stage parasitemia
TCP-2	(profile retired, see body of text)
TCP-3	Molecules with activity against hypnozoites (mainly *P. vivax*)
TCP-4	Molecules with activity against hepatic schizonts
TCP-5	Molecules that block transmission (targeting parasite gametocytes)
TCP-6	Molecules that block transmission by targeting the insect vector (endectocides)

Malaria is an infectious disease, and resistant parasite strains will always emerge, requiring the continual generation of new molecules. The last 4 years has seen a new generation of compounds with novel mechanisms of action entering clinical development [6]. These have resulted from a combination of phenotypic screening and rational design, with four new compounds currently shown to be active in patients: OZ439 [7], KAE609 [8], KAF156 [9, 10], and DSM265 [11]. However, in the same 4 years there have been increasing reports of multi-drug resistant malaria in the Greater Mekong Sub-region (GMS). The decreased artemisinin effectiveness is correlated with Kelch13 molecular markers [12]; in addition; there is resistance against partner drugs, such as mefloquine and piperaquine [13, 14]. It is not clear how long it will take for these resistant strains to have clinical impact in Africa [15] but this is a matter of great concern. The spread may be slowed by the successful deployment of triple combinations of artemisinin plus two partner drugs, and this is being actively explored. It is clear that all new medicines need to be active against known resistant strains, and future-proofed against emerging resistance to the highest achievable degree. Currently, all new candidate molecules are tested against a wide variety of both clinically resistant strains and laboratory-generated strains, and activity in these assays is a key requirement for moving forward. In the last few years several chemotypes have emerged against which it has not been possible to generate resistance in vitro, using cultures of 10^9^ parasites. Such resistance-proof ‘scaffolds’ will become an increasingly high priority. But given that the global parasite population emerging over a year approaches 10^19^ parasites, there is a need to be cautious about any extrapolation.

A focus on malaria *eradication*, rather than *control* requires prioritization of different types of medicines. The emphasis should be on breaking the cycle of disease transmission, rather than curing individual patients. Ideally these should receive a single-dose regimen to simplify implementation. Most importantly such medicines need to be safe and sufficiently tolerated to be given to the widest range of recipients, including infants and pregnant women. This is a continuum of benefit-risk, with one extreme being the potential to use treatments for subjects with asymptomatic infections, or even those with no detectable infection [16]. The combined challenge of having medicines that are (a) safe and well tolerated enough to be given to such a wide range of subjects; (b) effective enough to cure 95% with a single dose, and yet (c) suitable to be given to those with asymptomatic infections, is a formidable gold standard and there will inevitably have to be ‘trade-offs’. The early stages of malaria eradiation will require a differential deployment of currently registered medicines and development of new medicines for safe use in settings other than treatment of symptomatic malaria. There has been increased discussion about combining three or more active molecules to combat resistance. In addition, existing registered medicines, such as primaquine [17] and ivermectin, have been proposed as ways of breaking the transmission cycle [18].

A common platform for discussion of the ideal and minimally acceptable qualities of new medicines is important, given the 15-year time-frame from discovery to launch. However, the ‘goal posts’ do move. Over the last 4 years, much has been learned about what the potential of new molecules entering clinical development is, but also their limitations. There has been considerable progress in mapping out the strategy for malaria elimination in individual countries, and an improved understanding of the enormous challenges faced to eradicate the disease. Based on these insights, these profiles have been refined and updated in discussions with the wider malaria community. These refinements help to identify progress, and highlight key gaps that remain to be overcome to achieve this ambitious goal.

## Insights into new medicines for malaria indications

**Fig. 1:**
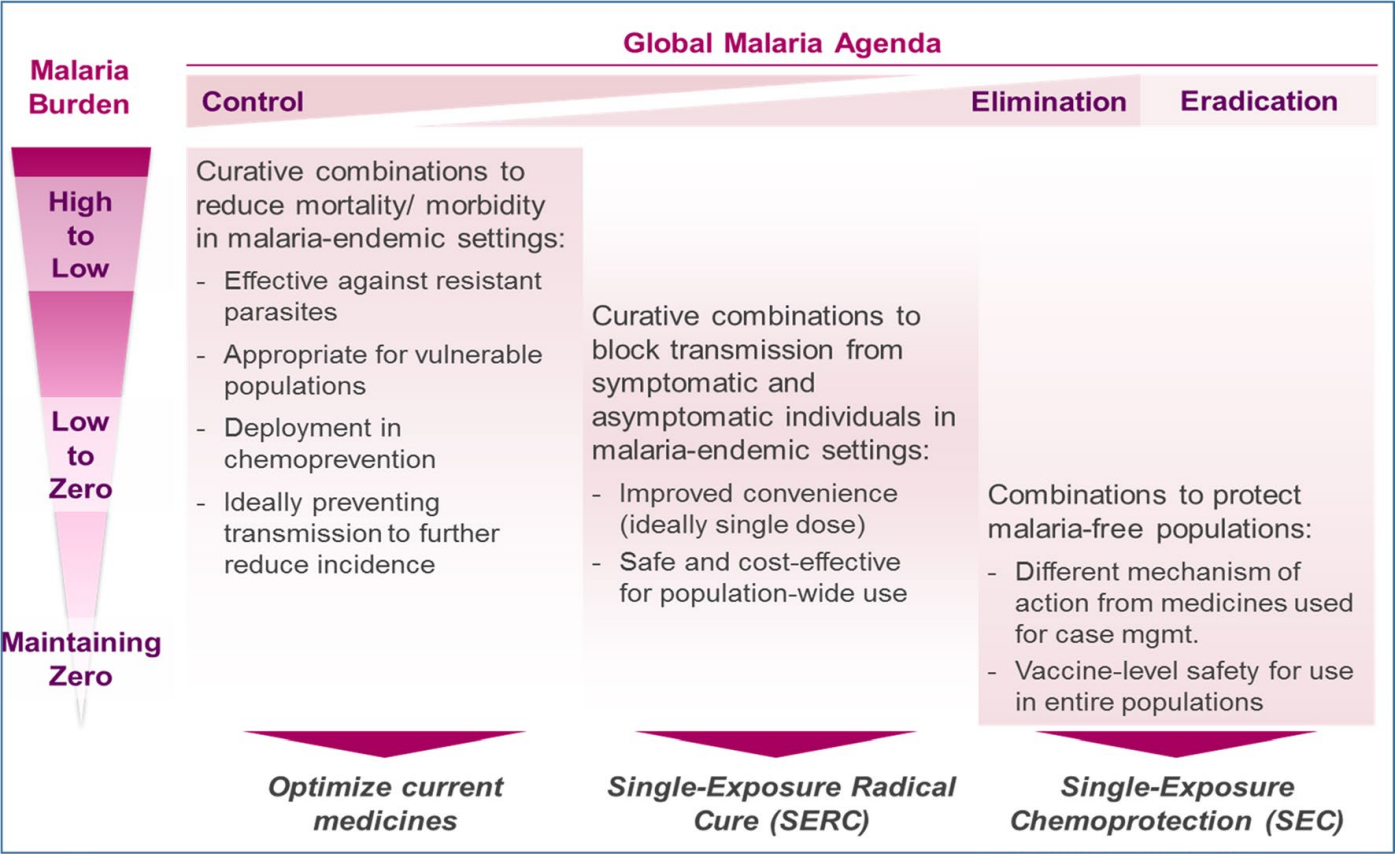
The role of current and new medicines in driving the reduction of malaria to zero and maintaining elimination in countries ([3, 175] and ‘malERA Refresh’, manuscript submitted). Given that even the most advanced new blood schizonticides will not be approved into policy until the 2020s, much of the initial phase of reduction will be carried out using current medicines, continually optimized for deployment. Transmission blocking will be achieved by the use of insecticides and other vector control methods. As resistance develops there will be a need for new classes of medicines, ideally capable of shortening the treatment course and simplifying therapy (labelled here as SERC, but also including two- or even 3-day regimens). For countries in pre-elimination and elimination, new classes of chemoprotectants will be needed, and this need will arguably increase as the number of countries in pre-elimination increases

The ideal medicine proposed in 2011 was a single encounter radical cure and post-treatment prophylaxis (SERCaP [2]). This would contain at least two active molecules, preventing the emergence of resistance in blood schizonticides. (The post-treatment prophylaxis is assumed to be largely from the blood schizonticides preventing new infections, and so the term is simplified to SERC). Over the last 4 years there has been increasing clarity on the role of different classes of medicines in elimination and eradication, both from WHO [3] and the UN Special Envoy for Malaria’s Aspiration to Action [19], which is summarized in Fig. 1.

The key role of new medicines for the medium term in this strategy is captured in this first TPP (TPP1; Table 2), for new medicines to treat malaria patients. In addition to the need to rapidly reduce parasitemia, the MalERA Agenda added the importance of preventing transmission, and of simplifying the therapeutic regimen from the current three to six doses, to a treatment that could be given after a single encounter with a health worker. These considerations remain a delicate balance to achieve (Fig. 5 in Ref. [4]). Over the last 4 years, a considerable body of data has become available on the safety of current medicines, in studies of tens of thousands of treatment events [20–22]. Detecting and de-risking projects from the potential occurrence of rare, life-threatening adverse events (AEs) requires extremely large clinical studies, and malaria-endemic countries do not provide strong self-reporting. This does underline the need for continued research using existing medicines; optimizing their uses depends on understanding the strengths and weaknesses of existing therapy. The critical issues are listed below.

**Table 2:** TPP-1 profiles for treatment of children or adults infected with malaria

Parameter to be demonstrated for the combination in clinical evaluation	Minimum essential	Ideal
Rate of onset of action	At least one component acts immediately; fever clearance at 24 h	Both components act immediately; fever clearance at 24 h
Proportional reduction in parasite load	Capable of achieving >12 log_10_ unit reduction in asexual blood-stage load; >95% patients free from parasites with from one to three doses	>12 log_10_ unit reduction in asexual blood-stage load in >95% patients with a single dose
Parasite-free (day 7), including patients from areas with known drug-resistance to current first-line medications	100%	100%
Clinical efficacy (ACPR at day 28 or later; per protocol)	>95% PCR-corrected, in a per-protocol population; on day 28; non-inferior to standard of care	>95% PCR-corrected, in a per-protocol population, on days 42–63; non-inferior to standard of care
Transmission blocking	Not required per se, but must not display detrimental drug–drug interactions with low-dose (0.25 mg/kg) primaquine	Combination should prevent clinical transmission, with no oocysts found in mosquitoes used in direct feeding or ex vivo experiments 15 days post treatment dose, without the need for low-dose primaquine
Relapse prevention: prevents the relapse of *P. vivax*, and by inference *P. ovale*.	Not required *per se*, but must not display drug–drug interactions with a relapse-preventing dose of 8-aminoquinoline	Confirmation in clinical studies (6 months in South America, Ethiopia and SE Asia, and the Pacific Ocean, potentially 24 months in India, Pakistan and Afghanistan)
Bioavailability/food effect	Predicted to be >30% for each molecule/ less than threefold (likely will be known by this stage)	Predicted to be >50% for each molecule/none (likely will be known at this stage
Drug-drug interactions	No unmanageable risk in terms of solid state or PK interactions	No risks in terms of solid state or PK interactions
Dosing regimen	Oral, two or three doses	Oral, once
Safety and tolerability	Few and manageable drug-related SAEs (serious adverse events), or adverse events leading to exclusion from study in phase III	No drug related SAEs; minimal drug-related AEs; no enhanced risk, no risk of hemolysis in subjects with reduced G6PD activity
Pregnancy	Not contra-indicated in second or third trimester	Not contra-indicated in second or third trimester, no suggestion of embryo-fetal toxicity in first trimester in preclinical species
Formulations	Co-formulated tablets or equivalent, with taste-masking (if needed) for pediatrics	Co-formulated tablets for adults. Dispersible or equivalent with taste masking (if needed) for pediatrics
Cost of treatment course	<$3.00 for adults and <$1.00 for infants younger than 2 year, benchmarked against the *most expensive* ACT ($3; ASMQ, artesunate/mefloquine)	≤$1.00 for adults, $0.25 for infants under 2 years, benchmarked against the *cheapest* ACT ($1)
Shelf life of formulated product (ICH guidelines for Zone IVa, b; combination only)	≥2 years	≥5 years
Susceptibility to loss of efficacy due to acquired resistance	Low: active against all known clinical strains. No evidence of transmissible resistant parasites in clinical development	Low: active against all known clinical strains. No evidence of transmissible resistant parasites in clinical development

## What clinical efficacy is required for a single molecule?

In 2013 the language that was used to characterize new molecules was arguably outdated: it listed TCP-1, for molecules that cause a fast reduction of the parasite load, similar to artemisinin, and TCP-2 for sustained anti-parasitic activity, as is seen with 4-aminoquinolines. In the last 4 years the characterization of the relative strength of blood schizonticides has improved, with concepts such as the total Parasite Reduction Ratio [23]. Clinically, an ideal schizonticide within a treatment should produce a sterilizing cure on its own. In monotherapy, all new compounds in clinical development show incomplete efficacy when given as a single dose, with an estimated PCR-adjusted per protocol adequate clinical and parasitological response (ACPR) at day 28) of 50–90% (the same range as marketed drugs). It is obvious that many compounds could achieve a complete cure by themselves, with repeated administration.

There is now a better understanding of the strength of the current molecules in the portfolio but because of their experimental nature, the early clinical studies of arterolane (OZ277 [24]) were limited to 6 h, while those of artefenomel (OZ439 [7]) and cipargamin (KAE609 [25]) were limited to 36 h after the last administration of the test drug, precluding an analysis of recrudescence later in time. But more recent studies in patients, such as those with a single dose of KAF156 [10], OZ439 [26], DSM265 [27, 28] or three doses of ferroquine [29] have allowed a 28-day follow-up. The best molecules are now giving clinical efficacy of 70–90% when used as monotherapy.

Prior to testing in human subjects, the best surrogate is to estimate the total Parasite Reduction Ratio PRR_tot_ [23] based on preclinical values. Estimations of minimum inhibitory and minimum parasiticidal concentrations (MIC and MPC) can be benchmarked against clinical observations [30]. Such translational pharmacokinetic/pharmacodynamic (PK/PD) modelling and simulation provide a much better framework for ranking the anti-malarial properties of molecules in the portfolio than the old bimodal fast-acting/short-duration versus medium-acting/long-duration classification.

## The single-exposure cure dilemma: efficacy versus safety in the wider population

Compounds that are used in combination to effect a single-encounter radical cure must be extremely potent and extremely well tolerated. When providing the total dose into a single administration with prolonged duration of effective exposure, there is an increase in the maximum plasma concentration, which places additional safety constraints. Preclinical safety evaluation and early studies in volunteers and patients can rule out compounds which are likely to have a poor tolerability profile, especially issues around nausea and vomiting. However, given the limitations of AE reporting after approval in malaria-endemic countries, the full picture of rare, serious AEs is a critical public health need. Any safety signal detected in studies prior to registration must be further evaluated in the field through large phase IV studies. The work over the last 5 years on artemisinin combination therapy (ACT) underscores the scale of this problem. For amodiaquine–artesunate, a study with over 15,000 malaria cases in Ivory Coast was required as part of the WHO-approved Risk Management Plan. Dihydroartemisinin (DHA)–piperaquine was linked to an increased QTc (corrected Q-T wave) interval but no further cardiac *sequelae*; gathering data from over 16,000 patients [31], and 10,000 patients, including a nested study of 1000 patients with thorough electrocardiogram (ECG) monitoring [32] has been required for this treatment to be accepted for WHO prequalification. Pyronaridine–artesunate (Pyramax^®^) produced an acute, transient and asymptomatic elevation of liver enzyme levels in phase III patients, also seen in healthy Caucasian volunteers after re-dosing. A study of over 13,000 dosing events was needed before a European Medicines Agency (EMA) recommendation could be made to allow repeated dosing without prior assessment of liver function tests [33]. Although malaria is treated as an acute infection, anti-malarials need to be safe enough to treat multiple infections. The trend of requiring additional data increases both significantly the timelines for a new medicine to enter policy guidelines, and impacts costs.

Showing a medicine is sufficiently well tolerated in populations with different risk–benefit profiles, such as those with asymptomatic infections [16], pregnant women [34], infants [35], and patients with other co-morbidities, such as human immunodeficiency virus (HIV) and tuberculosis (TB) co-infection, or malnutrition, adds complexity. One medicine may not serve all these groups. There is a time challenge in pregnancy; because of ethical issues of recruiting pregnant women into studies, such safety data is at present collected passively. It has taken two decades post-Stringent Regulatory Agency (SRA) approval to collect enough safety data to obtain a WHO recommendation allowing the use of artemether–lumefantrine in first-trimester pregnancy [36–40].

## The need to prioritize chemical scaffolds and combinations that are not prone to resistance generation

Although one can rank compounds based on their ability to generate resistant mutants in vitro, the understanding of how this translates to clinical resistance and the transmission of resistance is too limited to be used for decision making. Recent studies with compounds from the open-access Malaria Box [41] have identified several chemotypes against which resistance cannot be generated in the laboratory [42]. Attractive scaffolds from within this set will be prioritized for optimization and progress, and in parallel, target identification will be important.

## Matching pharmacological duration of cover

ACT has very poorly matched pharmacological durations of cover, principally because of the PK mismatch, with concentrations of artesunate dropping below the clinically active concentrations within 12 h of each of the three daily doses, compared to the partner drugs maintaining active concentrations for several weeks. All new pre-clinical candidates are now selected on the basis of being able to achieve active plasma concentrations for at least 4 and, in the best cases, 8 days. However, none of the new chemical classes, based on current knowledge, achieve the same coverage from a single dose as 3 days of treatment with 4-aminoquinolines or amino-alcohols [43]. Longer plasma exposure to provide greater post-treatment prophylaxis will always be needed; total protection from re-infection for 28 days or even later after treatment would clearly be preferable [44].

## Low variability of exposure and the absence of food effects are critically important

A combination that successfully treats at least 95% of patients must achieve adequate exposure in a minimum of 95% of patients who span a range of parasitemia burden and sensitivity. Therefore, reduced variability of exposure is important. Candidate molecules whose exposure is independent of food effects in humans are ideal. Food intake cannot always be controlled in the field, in particular the first day of treatment during the most acute phase of the disease, and the fat composition of food is highly variable, especially in low-income countries. These factors are even more important when considering a single-encounter cure rather than a 3-day regimen. Unfortunately, the absence of a food effect in preclinical development does not always predict the human situation and so this has to be verified clinically. Modelling of the variability of exposure in humans is a critical activity.

## More potent molecules are needed

Current clinical candidates have human dose predictions of between 30 and 1000 mg for a single, adult dose. It would be good to increase the stringency of selection here; compounds with doses <25 mg in infants and 100 mg in adults will be critical to reduce pill size, formulation volume, increase tolerability, and reduce the occurrence of vomiting. Compounds that require extensive formulation development are also problematic, especially since formulation adds considerably to the mass of the medication. The major cost driver is the quantity of drug in the treatment rather than cost per kilogram per se, thus more potent molecules would generally also lead to cheaper therapy.

## Combinations with two new chemical classes would be preferred

Four years ago, with no new molecules in later-stage development, the priority was to deliver *any* new medicine. This has led to phase IIb study proposals where new drugs were partnered with the best of the existing anti-malarials, typically piperaquine. This trend has now been tempered by a better understanding and appreciation of partner drug resistance. The current combinations in phase IIb (OZ439, KAF156) are partnered with molecules from known scaffolds, but selected to minimize the risk of cross-resistance with marketed and clinical-stage anti-malarials. For example, ferroquine is a new 4-aminoquinoline without cross-resistance to piperaquine [45]. Clinical resistance to lumefantrine is a matter of active current discussion, but appears not yet to be widely reported, and in vitro resistant mutants have not been identified [46]. In the coming 4 years the next-generation combinations entering phase IIb combination studies should contain two completely new chemotypes. Combinations of two or more new compounds are considered more complicated from a regulatory viewpoint. Putting together two new compounds is inherently more risky because the potential issues of the newer compounds are likely not yet evident. It may be that malaria mirrors drug development for other infectious diseases, with registration as a single agent (for use in combination with current therapy), but deployment as new combinations. This is a critical debate.

## Compounds ideally need to target more than one *Plasmodium* life-cycle stage

Although most of the current portfolio was originally identified from screening against asexual blood schizonts, many compounds have been shown to have activity in cellular assays for transmission-blocking (preventing the production of stage V gametocytes) as well as for chemoprotection (inhibition of developing hepatic schizonts). Any new combination must either have transmission-blocking efficacy itself, or be compatible with low-dose (0.25 mg/kg) primaquine, which is the WHO-approved regimen for transmission blockade. Considering the relapse of dormant *Plasmodium vivax* or *Plasmodium ovale*, no new scaffolds beyond 8-aminoquinolines have been identified with anti-hypnozoite activity and potential for development. In phase II studies, the 8-aminoquinoline tafenoquine has demonstrated high activity as a single dose [47]. A new candidate needs to have a similar efficacy to tafenoquine, but without the potential risk of hemolysis in patients with low glucose-6-phosphate dehydrogenase (G6PD) activity. New candidates are routinely tested in new animal models to monitor the risk of such hemolysis [48].

The fact that the new generation of compounds, when administered alone, cannot fully clear all asexual and sexual stage parasitemia with a single dose is not surprising, since none of the previous generation of therapy could. Going forwards, combinations in clinical trials may require multiple exposures, or single-exposure combinations of three or more entities to be successful in the vast majority of patients. Treatment with three or four drugs is becoming standard in other diseases such as TB [49], HIV/AIDS [50] and neonatal sepsis [51].

There has been increased discussion about the need to treat asymptomatic carriers, particularly in the context of disease elimination and eradication, to reduce the parasite reservoir [16]. Asymptomatic malaria causes significant co-morbidity through anemia [52]; however, it remains to be established if this risk on its own is sufficiently severe to justify the administration of a full treatment course. Moreover, the recent observation that the malaria parasite burden correlates positively with survival from Ebola infection will complicate this further [53]. Median parasite burdens may be lower in asymptomatic populations [54], but their range overlaps with the range found in uncomplicated symptomatic malaria patients (Kamija Phiri et al., submitted; the median baseline was 1240 parasites/ml and the range was 80–55,400/ml in asymptomatic subjects). Similar potencies are likely to be required to provide asexual-stage clearance in asymptomatics or symptomatics. However, in the context of elimination and eradication, the purpose of treating asymptomatics is principally important to decrease the parasite reservoir and reduce transmission. Indeed, elimination and eradication of a *Plasmodium* species will only be feasible via a comprehensive malaria treatment programme administering combinations to all, such as mass screen-and-treat (MST), seasonal malaria chemoprevention (SMC), or even mass drug administration (MDA). Treating asymptomatic or parasite-free healthy subjects will require higher degrees of safety, such as typically required for vaccines.

A key goal of treating asymptomatics is to not only eliminate the asexual parasites but also the gametocyte populations. With current compounds, effects on gametocytes are often weaker than on the asexual stages, and there is a clear need for compounds with specific transmission-blocking capabilities measurable in human transmission models. Understanding the activities of the current ACTs in asymptomatic malaria will be a key part of refining whether a new TPP is required for this indication.

## Insights into new medicines for reducing the incidence of malaria cases

The significant reduction in the incidence of overall malaria infection over the last 15 years has been achieved by protecting the at-risk populations, particularly with insecticide-treated bed nets [55] and spraying with insecticides and larvicides [56]. Vaccination represents another potential strategy, and although recent results with the RTS,S vaccine are encouraging, they are currently far from the level required to drive eradication alone [57, 58]. Other approaches such as radiation-attenuated sporozoite vaccines are under investigation [59]. Antimalarials offer an additional approach to reducing the incidence of malaria. The previous TPP proposal [4] had described single exposure chemoprotection (SEC) as the second TPP-2). The terminology is important here. This review uses *chemoprotection* to describe medicines used to protect subjects entering an area of high endemicity. In addition, in the final stages of malaria elimination and eradication, these could also protect populations at risk from emergent epidemics. *Chemoprevention*, in the sense of SMC, is used today to describe medicines with demonstrated efficacy for treatment at full treatment doses (because some of the subjects treated will be asymptomatic carriers), given regularly to large populations who live in areas of high endemicity.

**Fig. 2:**
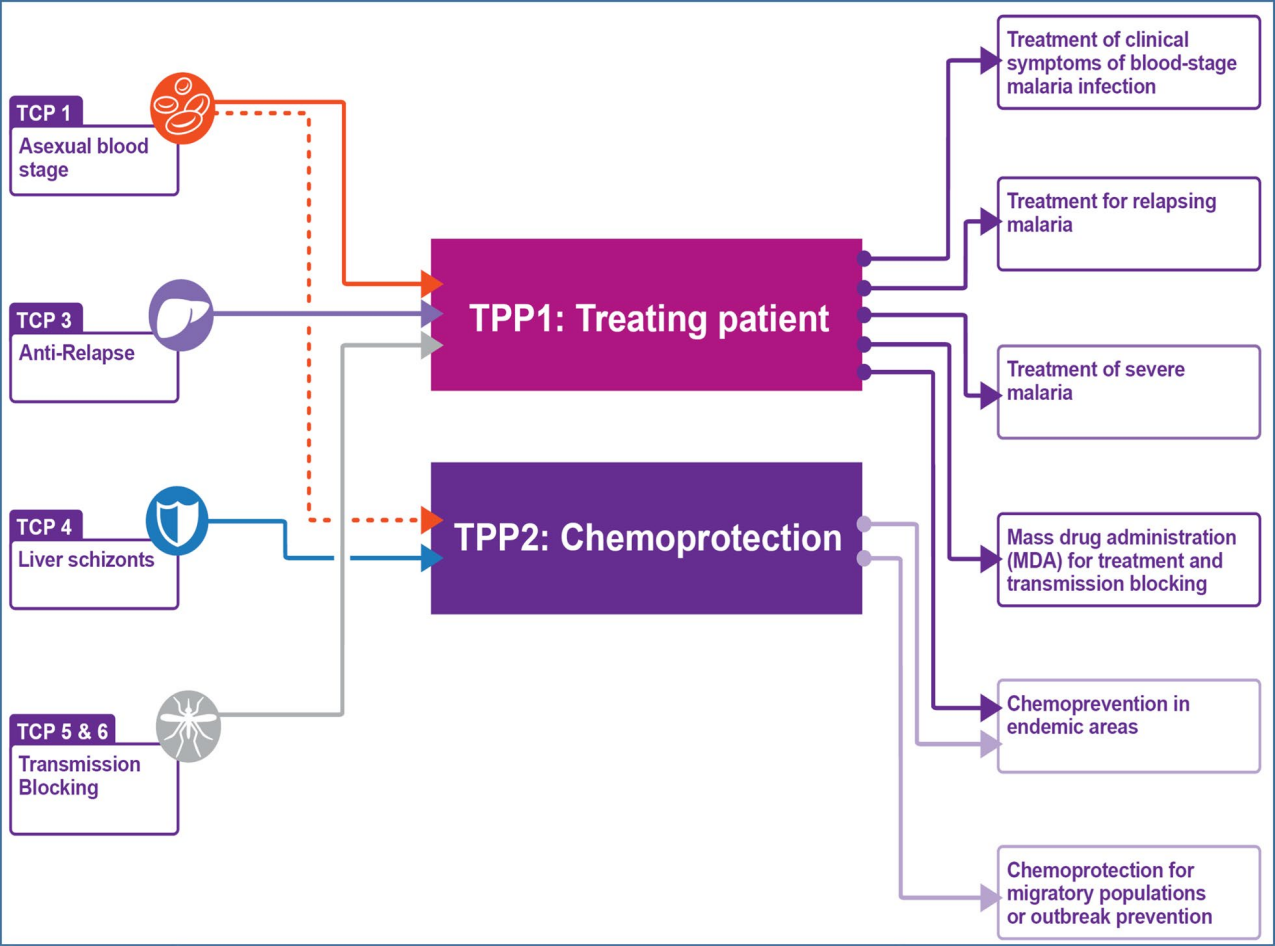
Inter-relationships between the two high-level target product profiles (*center*) with the individual target candidate profiles (*left*) for molecules that are part of the product. The uses for each product are summarized on the *right*

These two interventions play different roles at different times in eradication as illustrated in Fig. 2. Chemoprotection is a critical success factor later on for protecting migrant populations, and protecting static populations against new epidemics. Chemoprevention is most effective in high-transmission settings, and so is a critical success factor in the early stage of elimination. This review includes here a TPP-2 for chemoprotection, in addition to the TPP-1 for treatment (Table 3).

**Table 3:** TPP-2 chemoprotection profiles

Parameter to be demonstrated for the combination in clinical evaluation	Minimum essential	Ideal single exposure chemoprotection
Drug product	For elimination phases at least one of the two compounds also with TCP-4, co-formulated. The other should be a long-lasting blood schizonticide TCP-1	For elimination phases both molecules should have TCP-4 activity, co-formulated
Dosing regimen	Oral, once per week; injectable once per 3 months	Oral once per month; injectable less frequently than once per 3 months
Rate of onset of action	For asexual blood-stage action—slow onset (>48 h)	
Clinical efficacy	≥80% reduction in cumulative incidence of symptomatic malaria and non-inferior to Standard of Care	≥95% reduction in cumulative incidence and non-inferior to Standard of Care
Transmission blocking	No	Yes
Bioavailability/food effect	Predicted or measured >30% for each molecule/less than threefold	Predicted or measured >50% for each molecule/no significant food effect
Drug-drug interactions	No unmanageable risk in terms of solid state or PK interactions	No risks in terms of solid state or PK interactions
Safety and tolerability	Few and manageable drug-related SAEs in phase III and IV	No drug-related SAEs; minimal drug-related AEs that do not result in Study exclusion
Use in patients with reduced G6PD activity	Testing not required; no enhanced risk in mild-moderate G6PD deficiency	No enhanced risk
Pregnancy	Not contra-indicated in second or third trimester	Not contra-indicated in second or third trimester, no suggestion of embryo-fetal toxicity in first trimester in preclinical species
Formulations	Co-formulated tablets or equivalent, with taste-masking for pediatrics if taste is unacceptable to children Long-lasting formulations for intramuscular or intradermal use with low injection volume	Co-formulated tablets for adults. Dispersible or equivalent with taste-masking for pediatrics
Cost of treatment	≤$1.00 for adults, $0.25 for infants under 2 years Similar to vaccine costs for an injectable	Idem
Shelf life of formulated product (ICH guidelines for Zones III/IV; combination only)	≥2 years	≥5 years
Susceptibility to loss of efficacy due to acquired resistance	Very low; no cross resistance with partner	Very low; no cross resistance and orthogonal mechanism from those used in treatment

## Chemoprotection

A SEC medicine would optimally be a combination of two compounds, with minimal acceptable profile of causal liver stage activity, plus potential benefit from activity against asexual blood stages, which need not be fast-onset. The definition of this TPP, TPP-2, was conceptually built around atovaquone-proguanil (causal liver stage activity, and a profile tolerance for slow onset of action against asexual blood stages). It is an open discussion whether this needs to be a combination of two molecules, given that they face a relatively low burden of parasites. However, as soon as asexual blood-stage activity is included, there is an increased risk of selection of mutants, and pharmacodynamically matched combinations would be ideal. Frequency of administration is a critical question here; monthly, or even less-frequent dosing would be ideal, but weekly is still an option although it would have considerable consequences for implementation.

All the molecules in the malaria portfolio are now routinely tested for causal liver-stage activity, required for TCP-4, and many show good activity in vitro. However, the pharmacokinetics of these new molecules would only support a once weekly rather than once per month administration. Resistance generation for compounds with pure hepatic schizont activity is less of a concern, since the parasite burden is so much lower in the hepatic stages rather than the blood stages. However, new drugs should be active against pre-existing resistance mutations (including those to atovaquone). The pathway for regulatory approval still needs to be clearly defined and this will be a challenge over the next 5 years. Safety is a major concern as the drug would be administered to a broad population. The presence of long-duration blood schizonticide activity (TCP-1; Table 4) in one or more of the active ingredients in such a medicine may be an additional advantage for chemoprotection, since many subjects may have asymptomatic infections, and fluctuating parasitemia which may at times only be detectable by PCR. However, it does increase the risk of resistance generation.

**Table 4:** TCP-1 profiles, molecules that clear asexual parasitemia

TCP-1 criteria at human proof of concept	Minimum essential	Ideal
Dosing regimen; adult/pediatric dose	Oral, single dose (predicted) <1000 mg/<250 mg; oral, three doses <400 mg/<100 mg for areas of multidrug resistance	Oral, single dose (predicted); <100 mg/25 mg
Rate of onset of action and clinical parasite reduction ratio from single dose	Rapid clearance of parasites at least as fast as mefloquine (≤72 h from the highest burdens) and projected >10^6^-fold reduction in parasites	Immediate and rapid clearance of parasites at least as fast as artesunate; >Projected 10^12^-fold reduction in parasites
Susceptibility to loss of efficacy due to acquired resistance	No fit and transmissible drug-resistant parasites identified; identification of combination partner with no cross resistance	Very low (similar to chloroquine); no cross-resistance with asexual blood-stage combination partner. Resistance markers investigated
Relative clinical efficacy from patients in areas known to be resistant to current first line medications	Clinical efficacy against all known resistance (3-day dosing)	Clinical efficacy against all known resistance (single dose)
Drug- drug interactions	No unsurmountable risks with potential anti-malarial partners	No interactions with other anti-malarial, anti-retroviral or TB medicines
Safety	Therapeutic ratio >tenfold between therapeutic exposure and NOAEL (no adverse effects level) in preclinical studies, and easily ‘monitorable’ adverse event or biomarker for human studies	Therapeutic ratio >50-fold between therapeutic exposure and NOAEL in preclinical studies and easily ‘monitorable’ adverse event or biomarker for human studies
G6PD (glucose-6-phosphate dehydrogenase) deficiency status	Measured—no enhanced hemolysis risk from testing in SCID mice engrafted with human blood from volunteers with reduced G6PD activity; clinical confirmation	Measured —no enhanced hemolysis risk in subjects with reduced G6PD activity, with clinical confirmation
Formulation	Simple and inexpensive to produce, not requiring proprietary methodology or kits; can readily be produced in endemic countries	Simple and inexpensive to produce, not requiring proprietary methodology or kits; can readily be produced in endemic countries
Cost of active ingredient in final medicine	Similar to current medication: ≤$0.5 for adults, $0.1 for infants under 2 years	Similar to older medications: <$0.25 for adults, $0.05 for infants under 2 years
Estimated stability of final product under Zone IVb conditions (30 °C 75% humidity), in final packaging	≥24 months	≥3–5 years

The insights into the development of new medicines for chemoprotection over the last 4 years in this area can be summarized as follows.

1. Confirmation of the activity in humans for activity against hepatic schizonts or newly emerging asexual erythrocytes can now be tested in controlled human malaria infection (CHMI) volunteer studies using either injected cryopreserved sporozoites [60, 61] or infectious mosquitoes [62]. Currently these models involve infection of non-immune volunteers who live in non-endemic regions, and so it will be important to extend this work to include studies of the impact on the immune response, and in an expanded genetic background.
2. Historically, new medicines for chemoprotection have been destined for use by Western travellers. In an elimination and eradication campaign the priority population will be primarily travellers within Africa, travelling from areas of high endemicity to areas of low endemicity. In countries newly free from malaria, chemoprotection could also be used to protect the general population should they be at risk from epidemics. Molecules with chemoprotection activity should be tested on subjects with the correct ethnicity, and opportunities for this occur in people living in previously endemic zones, but now moving to endemic zones: for example, migrating from south to north Zambia, or west to east Gambia.
3. The most advanced malaria vaccine, Mosquirix (RTS, S-AS202) has been given a ‘positive scientific opinion’ by the EMA, but has sub-optimal and short-lived efficacy. The current course of vaccination involves three or four 0.5 ml intramuscular injections with a 25-gauge needle, at a cost of around $5 per injection. This has raised the possibility that small molecules could play a key role in long-term protection. An injectable sustained-release formulation would be acceptable if efficacy could be delivered by three or four injections per year. In HIV, where developing an effective vaccine has also been a major challenge, such an approach (called pre-exposure prophylaxis, or PrEP) is being studied [63], leading to the development of long-acting, nano-milled, injectable formulations aiming at 3 months or longer protection [64]. A TCP for an injectable chemoprotective agent for malaria can be developed (Table 5).

**Table 5:** Profiles for TCP-3, activity against hypnozoites (mainly *Plasmodium vivax*)

TCP3: general considerations	Minimum essential	Ideal
Dosing regimen; adult/pediatric dose	Oral, single dose (predicted) <1000 mg/<250 mg; oral, three doses <400 mg/<100 mg for areas of multidrug resistance	Oral, single dose (predicted); <100 mg/25 mg
Efficacy	In combination, prevents 80% of relapses^a^ over 6 months	In combination, prevents 80% of relapses over a year^a^
Safety and tolerability	Therapeutic ratio >tenfold between therapeutic exposure and NOAEL in preclinical studies and easily monitorable adverse event or biomarker for human studies	Therapeutic ratio >50-fold between therapeutic exposure and NOAEL in preclinical studies and easily monitorable adverse event or biomarker for human studies
G6PD deficiency status^b^	Therapeutic dose shows minimal differential change in hemoglobin concentration in mild and moderate G6PD deficient patients compared to patients with normal activity levels. New candidate drug shows no enhanced hemolytic risk in preclinical model	Therapeutic dose shows no significant change in hemoglobin concentration
Formulation	Acceptable clinical formulation identified which can be co-formulated with currently used blood schizonticides	Acceptable clinical formulation identified which can be co-formulated with currently used blood schizonticides

^a^Currently it is not possible to separate relapse from recrudescence of *P. vivax*. The ideal efficacy of 90% is based on the values seen for both primaquine and tafenoquine in its phase II study

^b^The current benchmark would imply a lower degree of hemolysis in severely G6PD-deficient patients, with no additional safety signal requiring monitoring in the field

## Chemoprevention

Chemoprevention is defined primarily in the context of current thinking on SMC [65] and related strategies. SMC is currently defined as the intermittent administration of full treatment courses of an anti-malarial medicine during the malaria season, to prevent malarial illness. The objective is to maintain preventative anti-malarial drug concentrations in the blood throughout the period of greatest malarial risk. The high efficacy of SMC campaigns with sulfadoxine-pyrimethamine plus amodiaquine [66–70] highlights a potential wider role for this approach in the future, possibly in combination with vaccination. Concerns over sulfadoxine-pyrimethamine resistance in Africa south of the Equator [71] has led to trials of monthly 3-day courses of ACT in chemoprevention [70], contrasting with the WHO recommendation to use different medicines for chemoprevention and treatment [72]. This review does not propose a TPP for chemoprevention. The likely time-lines for the development of such a medicine based on new chemical entities would take too long for it to be useful in the elimination agenda. The reality is that chemoprevention in high-transmission zones will continue to use the drugs that are currently available.

The regulatory pathways for new medicines in chemoprotection and chemoprevention still need to be clarified: SRAs may require a clearer basis for the dose selection than that used previously for atovaquone-proguanil and mefloquine. An updated draft strategy (Macintyre et al., unpublished) would establish an understanding of drug exposure needed for chemoprotection and chemoprevention in addition to that required for curative treatment, as these exposures may differ.

## Severe malaria

In severe malaria, patients are often unconscious or otherwise too sick to take oral medication. At this stage of disease, the sequestration of large numbers of parasites in the microvasculature of the brain leads to brain swelling [73], and in some cases affecting the lungs and other vital organs, which may rapidly lead to death, whereas in young children the major complications may result from severe anemia. Any new medicine would therefore have to free up the microvasculature, either by disengaging or killing the parasites; the drug’s speed of onset of activity is therefore of paramount concern. The AQUAMAT and SEAQUAMAT studies that compared intravenous artesunate and intravenous quinine [74, 75] and other studies that compared intravenous or intramuscular artesunate [76, 77] showed that injected artesunate reduces the overall severe malaria patient mortality by between a third and a quarter. From a safety viewpoint, quinine use has been associated with injection-site pathologies, cinchonism [78, 79] and also risks of hypoglycemia. Artesunate, as used in severe malaria, has been linked with late-onset hemolysis [80–82], with the associated increased clinical risk, and it would be important to establish early on if this is a general issue for all fast-acting compounds. Any ideal next-generation molecule would clearly not have these disadvantages. Artesunate suppositories have been proposed for pre-referral of severe malaria [83], specifically for children under 6 years old who are more than 6 h away from a hospital, but this formulation also may offer an approach to severe malaria when intramuscular injection is not possible. Simpler, pre-filled injection devices would be useful, but have to contend with the instability of artesunate in aqueous media.

Artesunate resistance has been a concern in the GMS for almost a decade, and parasite reduction half-lives of 12–15 h have been reported [84], representing a four- to six-fold reduction in speed of kill compared with the treatment of African patients. Ultimately a new fast-acting compound may be needed to treat severe malaria. The development route for a new product targeting severe malaria would not be easy [85], since adult cases of severe malaria are rare, and proceeding directly to pediatric patients would be ethically difficult. Any pivotal trial would need to be large; for example, artesunate required data from over 6000 severe malaria cases [74, 75]. If artesunate fails for severe malaria, any new drug would have to perform better than injectable quinine, which would revert to being the gold standard. One starting point would be the further development of the rapid-onset TCP-1 molecules (as such TPP for severe malaria is a product using a molecule from a sub-set of the TCP-1 portfolio). Since many of these are relatively hydrophobic, this would require the development of a parenteral formulation as a first step. Amongst the current portfolio KAE609 clears parasites more rapidly than artesunate and could have value in severe malaria if a parenteral formulation were available [8]. Other PfATP4 inhibitors are in development, with SJ733 (+−SJ00557733; [86, 87]) recently starting phase I studies. A concern with targeting PfATP4 is that mutations in this gene confer resistance against aminopyrazoles, dihydroisoquinolones and spiroindolones [42, 88, 89]. The developmental pathway could be to demonstrate safety and tolerability of a parenteral formulation whilst assessing activity in adults with moderately severe malaria (hyperparasitemic cases; [85]).

Facilitating rapid de-sequestration has also been suggested as a therapeutic mode of action for severe malaria, with recent clinical testing of the anti-rosetting compound sevuparin [90]. One could hypothesize that a compound that blocks sequestration keeps parasites in the vasculature and thus increases the ability of both the spleen and the immune system to clear these. From a safety perspective, the concern would be to show that rapid de-sequestration did not lead to splenic overload from released, but damaged erythrocytes. Currently no candidate profile has been developed.

Approaches to reduce neurological *sequelae* or modulating the innate immune response have not advanced much. The conventional path forward would be to test medicines with a known pediatric safety record as adjunct therapy along with parenteral artesunate. However, the success rate to date has been extremely poor [91]. Perhaps the availability of standard neurological assessment technologies, such as magnetic resonance imaging (MRI) in malaria-endemic regions [92] will provide new insights. The safety/efficacy equation is somewhat different for severe malaria, due to the high risk of death; a rapid onset of action is the most critical factor. It is to be noted that many preclinical animal models for severe malaria have been reported and used for compound testing but, unfortunately, the validation of these models is poor, simply because compounds have been unable to be taken forward for confirmation in humans. Critically, it is only data in humans and ultimately children that could provide sufficient validation to justify further clinical work. Recently genetic and experimental data were obtained that suggest that elevated levels of angiotensin II may protect from mortality due to cerebral malaria [93]. If true, this may provide opportunities for adjunct treatments for severe malaria.

## Selecting medicines for use in pregnancy and small children

Accelerating the safety assessment of new medicines in pregnancy is extremely challenging. The history of the assessment of artemisinin in first-trimester pregnancy shows how difficult the path is. Although preclinical safety signals were seen in many experimental species, no overt increase in birth defects or early abortions have been seen with artemisinin use in humans. To establish that there was not a twofold increase in the risk of such an event, an analysis of some 800 reported inadvertent ACT exposures in first-trimester pregnancy was needed [39, 94, 95]. For any new medicine, it would usually be at least a decade after launch before such data were available, underlying the need for more investment in the post-registration safety monitoring in disease-endemic countries. In the meantime, the best approach is at least to triage new compounds for any potential risk factor in early pregnancy by bringing forward the standard regulatory examination of early embryo-fetal development (EFD) toxicity. This is conventionally done in parallel with phase II, but MMV is now routinely performing this ahead or in parallel with phase I, and plans to make it part of its standard preclinical safety assessment. Furthermore, within discovery, MMV is exploring state-of-the-art in vitro and in vitro models for assessing and predicting reprotoxicity and teratogenicity, including (non-human) whole embryo cultures, embryonic stem cells and zebra fish embryos. Unfortunately, none of these methods has yet reached the level of confidence in predictive value that has been achieved for in vitro genotoxicity assays such as the Ames assay, and thus the earliest regulatory-relevant assays remain the rat and rabbit EFD studies. An alternative approach to small molecules would be the use of monoclonal antibodies in this patient population, since the off-target liabilities of monoclonal antibodies are accepted to be lower than for small molecules.

Symptomatic malaria infections are particularly common in small children and infants in disease-endemic countries. The main reason is the lack of a protective immune response. During clinical development it is therefore important to verify early on that medicines are effective in populations from countries with low malaria endemicity, to ensure that the medicine can be fully active without immune support. For pediatric medicines, it is important to fully understand compound exposures and the relationship between exposure and efficacy (PK/PD) in small children and then to adapt dosing accordingly. Typically the clinical studies have included children as young as 6 months, or 5 kg in weight. Between 6 months and 2 years old, the development of the liver and the re-distribution of fat in the infant often translates into substantially different exposures for similar dosing [96]; the International Conference of Harmonization (ICH; [97]) has recommended to divide childhood into five phases with respect to clinical drug use, reflecting this complexity. But only adults can be enrolled in clinical trials prior to phase II studies. One of the goals of the next few years is to better model the factors that control drug uptake, distribution and metabolism in small children. The other concern is safety, and it should be possible to accelerate preclinical juvenile toxicology studies to get an early read-out as to whether new compounds have any specific safety concerns.

## Linking the TPPs to individual molecules and TCPs

Both high-level TPPs require a combination of biological activities, and each of these can be defined by a TCP. The nuance is that a single molecule can achieve more than one TCP, for example having asexual blood-stage activity (TCP-1) plus transmission blocking (TCP-5). In total, any new combination medicines must have sufficient of these activities to achieve the TPP. This framework helps to define gaps in the current TPPs that are currently under discussion. For example, in the vaccine community, vaccines purely targeting the transmission of *Plasmodium falciparum* are under development, targeting Pfs25 [98–100], Pfs230, Pfs48/45 or (*Anopheles*) AgAPN1 [101]. Currently no such medicines exist, since all the molecules in development that have transmission-blocking activity on the parasite also impact the blood stages. However, with the increased attention to molecules blocking transmission through endectocide activity, there is discussion on the inclusion of a TPP for this activity, which could be a mixture of TCP-6 and pure TCP-5 molecules. Severe malaria products could also be included in this chart, as the sub-set of fast-acting TCP-1 molecules that are suitable for parenteral formulation.

## Insights into the evolution of TCPs

### Proposed changes to the nomenclature

Although this review’s description of TCPs helped develop a common language in the development of leads and preclinical candidates, there are many areas where it can be improved. As discussed above, for the blood schizonticides, the separation of compounds with rapid effect or long duration of action (TCP-1 vs TCP-2), is a historical artefact. Compounds in development have all been selected for long duration (a predicted pharmacologically active plasma exposure in humans of more than a week), as well as either fast or very slow parasite reduction. A more useful way of comparing compounds is by combining the speed of kill and PK into a total Parasite Reduction Ratio (PRR_tot_; [23]), giving a numerical index to the overall ‘strength’ of the compounds. These two profiles have now been merged into a single, new TCP-1 focused on clearance of asexual blood-stage parasites, and the TCP-2 nomenclature has been retired (Table 6). Medicines for severe malaria would be a sub-set of this TCP-1, with rapid onset of action and where a suitable parenteral formulation can be developed. (With the focus in the last 4 years on registering artesunate for injections and suppositories, this has not been a priority, but in the future new parenteral formulations of fast-acting compounds will become more important.)

**Table 6:** TCP-4 profiles, as part of chemoprotection

TCP-4: general considerations	Minimum essential	Ideal
Dosing regimen; adult/pediatric dose	Oral, once per week; <500 mg/<100 mg in infants. Simple oral formulation Injectable: subcutaneous or intra-muscular, once per month, with injection volumes <0.5 ml for infants via a 25 gauge or smaller needle	Oral, once per month; <100 mg, Injectable: subcutaneous or intramuscular once per 6 months
Susceptibility to loss of efficacy due to acquired resistance	No fit drug-resistant parasites identified in controlled human challenge model; no cross-resistance with partner drug in combination	Very low; no cross-resistance with partner drug in combination; independent mechanism to those of treatments used in geographical area
Clinical protection from symptomatic infection	>95% protective efficacy (positive parasitemia)	>95% protective efficacy (positive parasitemia).
Bioavailability/food effect—human data	>30%; no unmanageable food effect	>50%/no significant food effect
Drug-drug interactions	No unmanageable risks	No interactions with other anti-malarial, anti-retroviral or TB medicines or oral contraception
Safety and tolerability	Therapeutic ratio >tenfold between therapeutic exposure and NOAEL in preclinical studies and easily monitorable adverse event or biomarker for human studies	Therapeutic ratio >50-fold between therapeutic exposure and NOAEL in preclinical studies and easily monitorable adverse event or biomarker for human studies
G6PD deficiency status	Therapeutic dose shows minimal change in hemoglobin concentration in subjects with reduced G6PD activity. New candidate drugs shows no enhanced hemolytic risk in preclinical model	Measured—No enhanced risk in subjects with reduced G6PD activity
Formulation	Simple and inexpensive to produce, not requiring proprietary methodology or kits; can readily be produced in endemic countries	Simple and inexpensive to produce, not requiring proprietary methodology or kits; can readily be produced in endemic countries
Cost of single treatment	≥$0.5 for adults, $0.1 for infants under 2 years per month for oral protection; injectable could be priced to vaccine levels	<$0.25 for adults, $0.05 for infants under 2 years for oral treatment
Projected stability of final product under Zone IVb conditions (30 °C, 75% humidity)	≥3 years	≥5 years

The description of TCP-3a and 3b, originally based on activities of primaquine against the extra-erythrocytic forms has been confusing. TCP-3 has now been re-defined as the activity against hypnozoites directly (or indirectly through pathways such as apoptosis or autophagy) [102], or forcibly re-activating these so that, once metabolically active, they can be killed by a partner drug, analogous to the dormant HIV ‘shock and kill’ strategy [103, 104]. TCP-4 describes the attributes for activity against hepatic schizonts, as part of chemoprotection.

TCP-5 replaces the previous TCP-3b and describes molecules with transmission-blocking activity. This is one area where understanding has developed considerably over the last 4 years. The clinical reference for this is low-dose primaquine [105, 106]. Ideally, a candidate would have activity against all five differentiated forms of gametocytes (stages I–V), plus inhibition of oocyst or sporozoite formation in the mosquito vector. For *P. vivax,* gametocyte differentiation is coincident with asexual blood-stage proliferation and since all compounds that affect asexual vivax malaria appear to also kill vivax gametocytes, no special focus is required in this area [107]. Direct screening methods to find a transmission-only molecule have now been described [108], with the potential advantage of finding molecules that are less susceptible to resistance, however their clinical development path is more difficult [109]. The role of endectocides in transmission blocking has received much attention [18, 110], and the initial framework for a corresponding TCP-6 has been introduced. There are ongoing discussions at the WHO, which will further refine this over the next 12 months.

Establishing new, individual TCPs for anti-relapse (TCP-3; Table 7) and transmission blocking (TCP-5; Table 7) also re-emphasizes the critical importance of these two TCPs for elimination and eradication of *Plasmodium* spp, and the progress made since the last publication in defining appropriate drug discovery cascades [111]. The profiles are described in more detail below. Each describes a set of attributes for a single compound, for which there should be increasing confidence as the compound moves from lead (active in animal models [112]) through selection as a candidate drug, preclinical evaluation and phase I(a) studies to a demonstration of activity in humans in either CHMI models [26, 27, 30, 43, 113, 114], classically described as phase Ib, or early exploratory monotherapy patient studies (described as clinical exploratory, or phase IIa). Each TCP details a ‘minimum essential’ and an ‘ideal’ profile. The ‘ideal’ criterion builds on what is described in the ‘minimum essential’; criteria are not repeated unless there is a change.

**Table 7:** TCP-5 profiles, molecules with transmission-blocking activity

TCP-5: general considerations	Minimum essential	Ideal
Dosing regimen: adult/pediatric dose	Oral, single dose (predicted) <1000 mg/<250 mg; oral, three doses <400 mg/<100 mg for areas of multidrug resistance	Oral, single dose (predicted); <100 mg/25 mg
Efficacy	Prevents transmission prevalence to mosquito >90% in appropriate clinical protocol	Prevents transmission prevalence to mosquito >90% at 15 days post oral dose
Safety and tolerability	Therapeutic ratio >tenfold between therapeutic exposure and NOAEL in preclinical studies and easily monitorable adverse event or biomarker for human studies	Therapeutic ratio >50-fold between therapeutic exposure and NOAEL in preclinical studies and easily monitorable adverse event or biomarker for human studies
G6PD deficiency status	New candidate drug shows no enhanced hemolytic risk in preclinical model, and no concerns for severe G6PD deficiency	
Drug-drug interactions	No unmanageable risks	No interactions with other anti-malarial, anti-retroviral or TB medicines
Formulation	Simple and inexpensive to produce, not requiring proprietary methodology or kits; can readily be produced in endemic countries	Formulation without complex excipients possible; simple and inexpensive to produce, not requiring proprietary methodology or kits; can readily be produced in endemic countries
Cost of single treatment	Similar to current medications for asexual stages: $0.50 for adults	Similar or better than current transmission-blocking low dose primaquine <$0.05 for adults, $0.01 for infants for transmission-blocking
Projected stability of final product under Zone IVb conditions (30 °C, 75% humidity)	≥36 months	≥5 years

## TCP-1: ‘asexual parasite clearance’, reducing the parasite burden

The management of malaria cases requires the complete elimination of asexual parasites. The current combination treatments can achieve a cure rate (ACPR) assessed 28 or 42 days after treatment, in more than 95% of the (per protocol) population. How much drug is required, and how effective it needs to be to reduce parasitemia to below detectable levels in 19 out of 20 subjects will include factors such as the patient’s initial parasite burden, variability of drug exposure and immune status. However, for comparing molecules, the two key factors are the rate of parasite reduction (PRR) and the time over which an efficacious plasma concentration can be achieved [115]. These can be integrated to give a PRR_tot_ [23], as a measure of the power of an individual molecule. The rank order of PRRs can be estimated in vitro [116, 117], or in SCID (severe combined immunodeficient) NOD-SCID IL2Rδ^−/−^ mice [118]. Molecules can be initially ranked as very fast (such as PfATP4 inhibitors), fast (artesunate and other endoperoxides) and medium (as fast as mefloquine). These initial estimates can then be confirmed in CHMI models [26, 27, 30, 113, 114, 119] and other pilot clinical studies [7]. The estimates of the MIC and MPC from the SCID mouse model appear to correlate well with the values seen in humans [30]. The human treatment dose can be simulated for single or multiple doses by combining these data with the predicted human PK using standard methods that rely on preclinical in vivo PK and in vitro metabolism data. Studies with KAE609 in *Plasmodium berghei*-infected mice confirmed that efficacy correlated best with the area under the curve (AUC), i.e., an integration of exposure over time, or time with exposure above threshold level, rather than purely to C_max_. Further studies using the SCID mouse model, using dose fractionation, will be important going forwards to determine if this is general the case. One notable exception already identified is artesunate, where the plasma residence time is much shorter than the duration of the parasite lifecycle, and the efficacy is more simply linked to its C_max_ [120, 121].

An ideal TCP-1 compound, based on this review’s new definition, should be able to reduce parasitemia (PRR_tot_) 10^12^-fold by itself, based on curing adult patients with as high as 200,000 parasites/ml. This would hold for patients at the lowest fifth percentile of the predicted human plasma exposure variability. Setting preclinical criteria to discontinue a compound is difficult because of the lack of precision in PRR_tot_ estimates. These are highly dependent on the accuracy of terminal half-life estimates, which are exponentially related; a twofold longer half-life will cause a squared increase in the fold parasite reduction. This review pragmatically set the rule of thumb in discovery of 10^6^-fold reduction from a single dose. Also important is the shape of the concentration–time curve in humans. A low peak-to-trough ratio is desirable in order to minimize problems in safety and tolerability and maximize the duration of efficacy (i.e., the time above MPC) at the same time as limiting C_max_-related safety issues.

It remains under discussion as to whether there is a minimum rate-of-reduction criterion in parasitemia. The previous publication [4] focused purely on the rate of reduction, using 4-aminoquinolines as the benchmark. Experimental evidence shows that piperaquine has a 1og_10_ PRR over 48 h of around 3.4, or a parasite reduction half-life of 4.2 h [122]. Similar rates of parasite reduction have been reported for chloroquine [123] placing some 4-aminoquinolines as relatively fast killers clinically, with similar kinetics of parasite reduction as artesunate. Thus, the earlier criterion may have been too stringent; mefloquine and ferroquine for example, are an order of magnitude slower [30, 43]. The onset of action (lag phase) on parasitemia appears less important provided that, as a minimum, growth is immediately halted and then followed by rapid parasite clearance. Pyrimethamine, for example, is clinically highly effective on sensitive parasites despite having a delayed onset of action of approximately 24 h, because it subsequently clears parasites quickly and has a long human plasma half-life of 96 h. However, compounds with a long fever clearance time may present compliance challenges and safety concerns, especially in children.

Finally, TCP-1 molecules should ideally show good activity against the blood stages of all five *Plasmodium* species that infect humans. The difficulty of comparing data across species in in vitro assays comes from the fact that only *P. falciparum *is widely cultured, while most of the other *Plasmodium* species are tested ex vivo. Whilst the testing of preclinical candidates on field isolates (*P. falciparum* and *P. vivax*) or laboratory-adapted strains (*P. falciparum* and *Plasmodium knowlesi*) is becoming increasingly feasible, access to *P. ovale* and *Plasmodium malariae* parasites is extremely limited and, thus, activity against these two species is usually assumed rather than measured. Only in the case of pyronaridine-artesunate has activity against other species (*P. vivax*) been supported by clinical data in the stringent regulatory filing [21, 124]. The consensus is therefore that ‘ideal’ new molecules should have clinical activity demonstrated against *P. falciparum*, and comparable efficacy against other species in vitro, where obtainable.

As noted earlier, there is no specific TCP for severe malaria, since such compounds are a discrete sub-set of TCP-1 provided they produce appropriate immediate and rapid parasite clearance, are well tolerated and can be developed as an injectable. Compounds with a slow onset of action against blood stages, such as the macrolide antibiotics, have potential to play a role in chemoprotection, as explained in TPP-2 above, but only with safety, potency and PK to support dosing once per week or less often.

## TCP-3: targeting *Plasmodium* hypnozoites

The definition of a radical cure is the removal of all forms of the parasite from an infected individual, not just the asexual stages that cause symptoms. Whilst this definition covers all non-asexual blood stages, the focus of TCP-3 is on the form that is the hardest to kill: the dormant liver stages that follow new *P. vivax* and *P. ovale* infections. To be effective in vivax and ovale endemic regions, any new anti-malarial drug combination requires a molecule which can either kill the dormant hypnozoites directly, or through host-mediated pathways such as apoptosis or autophagy, or re-activate them, allowing them to be killed by other molecules in the combination. The gold standard for this profile remains primaquine, which is effective against *P. vivax* and, presumably, *P. ovale* hypnozoites. Primaquine has three weaknesses: compliance is poor, given the 14-day therapy course in asymptomatic individuals; gastro-intestinal tolerability; and the increased risk of hemolysis in patients with reduced G6PD activity.

Progress towards finding new TCP-3 molecules has been extremely challenging and hampered by the lack of cellular assays that directly measure hypnozoite inhibition. Until recently the gold standard was the double surrogate of in vitro infection by *Plasmodium cynomolgi* sporozoites of rhesus hepatocytes [125]. This links directly to the current gold standard in vivo primate model [126], but with the disadvantage of the species difference of both parasite and host cell.

Hypnozoite assays using primary human hepatocytes in co-cultures or spatial confinement [16, 127], or human cell lines [128, 129] have been established recently, and are now suitable for testing in either eight-, 96- or 384-well formats. However, supply of sporozoites for infection remains a major limiting factor for going forwards [130, 131]. Importantly, statistically useful measures of inhibition are now being obtained in 96-well and 384-well formats. These allow functional activity of hypnozoites and schizonts to be monitored and even in vitro relapses to be observed [132]. The exact protocol for compound incubation is important, given the time required to develop particular parasite forms. Significant activity in such assays is hard to define, since primaquine is relatively inactive in vitro, depending on the metabolic activity of the cells [102, 133]. Pragmatically, a threshold for in vitro activity of EC_50_ < 100 nM against hypnozoites has been set, although less potent compounds with acceptable safety profile at high plasma exposure would also fit the bill. New in vivo models that use human liver-chimeric mice have demonstrated infections with *P. vivax* sporozoites, formation of hypnozoites and liver schizonts, providing potential new preclinical models [134], although these are still a long way from routine use.

New clinical models [135] and clinical trial designs [136] mean that the anti-relapse potential of new clinical agents can now be measured reliably in humans, since, strictly speaking, the predictive power of the primate model has only been demonstrated for 8-aminoquinolines. The success of the tafenoquine phase II trials [137] underscores that it is possible to obtain definitive clinical data on relapse at 6 months, establishing a clinical proof-of-concept benchmark for this compound class. Tafenoquine does have the liability of hemolysis in subjects with reduced G6PD activity, and so the ideal next generation compound would be one that did not require G6PD monitoring.

## TCP-4: targeting hepatic schizonts

The terminology here has often been confusing, and it is important to repeat the definitions made earlier. Chemoprotection, as part of the SEC, is the use of medicines to protect subjects entering high-transmission zones from an area without malaria transmission, or to protect those in malaria-free areas at risk of epidemics in the final stages of malaria eradication. (This is distinct from chemoprevention, as in SMC, which is the use of full courses of blood schizonticides to prevent clinical malaria in populations that reside in endemic areas). This review uses the term TCP-4 activity to describe hepatic schizonticide activity. Historically, medicines that provide chemoprotection had erythrocytic schizonticide activities (TCP-1) as illustrated by atovaquone-proguanil (with TCP-1 and TCP-4 activity), or even compounds such as mefloquine, that is exclusively a TCP-1 compound. However, in designing new chemoprotectants, it is clear that the research community needs to proactively search for molecules with hepatic schizont activity.

No compound with pure TCP-4 activity currently exists within the global malaria portfolio: all the current compounds are dual-active TCP-1/TCP-4. However, new approaches to in vitro screening directly against hepatic schizonts using murine (or ideally human) malaria may change this situation [129]. In addition, murine models using sporozoites of luminescent parasites allow whole animal-imaging of parasites to determine efficacy [138], although mouse models that support development of human-relevant parasites are, of course, more relevant (for example the human liver-chimeric FRG KO huHep mouse model supports *P. falciparum* infection [139]). Several new scaffolds have been identified with activities against *P. berghei* or *P. falciparum* liver schizonts, including the phosphatidylinosine-4-kinase (PI4K) and dihydroorotate dehydrogenase (DHODH) inhibitors, and these are also prophylactic in in vitro assays of *P. cynomolgi* liver schizonts [126, 140]. Guidelines on the cellular efficacy are still empirical, and largely based on the experience with erythrocytic schizonticides, with a threshold activity for candidates of EC_50_ < 10-100 nM, and predicted human PK allowing the minimum prophylactic concentration (assessed in vivo) to be maintained for a week from a single oral dose. Again, empirically, this translates into fully protective doses in murine models with oral administration at <10 mg/kg. Activity can be confirmed in CHMI sporozoite challenge studies [60–62], and this will help put more objective criteria on TCP-4 over the coming years.

One important, emerging consideration is that TCP-4 can be expanded to include molecules intended for intramuscular or subcutaneous administration. Experience with vaccination helps define the profile of injection volumes below 0.5 ml, preferably subcutaneous, using a 25 or higher gauge needle, with activity lasting at least 3 months. Compounds used should have a relatively low susceptibility to resistance generation; breakthrough strains following intramuscular injections of cycloguanil pamoate in children were all resistant [141, 142], presumably due to pre-existing dihydrofolate reductase (DHFR)-resistant parasites in the field. The particle size and viscosity of the vehicle will be important; penicillin-G benzathine as a long-acting antimicrobial is precluded from routine pediatric use because of the required needle size [143]. Potency is important too; in psychiatric disease, drugs suitable for injection have oral adult doses in the 10-mg/day range, and this serves as a useful guide to early candidate identification for the long-acting formulation of an anti-malarial. Certain properties, such as poor aqueous solubility may now provide an advantage in maintaining long-lasting reservoirs, if they can be linked to high solubility in lipid vehicles and slow, controlled release from the depot. The safety challenges remain enormous: long-term safety (6-month exposure), and a cautious approach to human volunteer studies would be needed, especially for new molecules that have never been tested in humans previously. Such studies will necessarily be lengthy.

## TCP-5: transmission blocking

One of the key points where the parasite lifecycle can be broken is to prevent transmission from the infected human host to the mosquito vector. ACT does not block transmission, so the current WHO recommendation [72] is to use a single, low dose of 0.25 mg/kg primaquine to reduce transmissibility of treated *P. falciparum* infections in low-transmission areas. Higher doses are more effective but their use is limited by concerns over safety in subjects with reduced G6PD activity. Results from a recent study resulted in a proposal to dose primaquine by age group [144]. The clinical activity of low-dose primaquine sets the bar for the next generation of compounds. Primaquine must be metabolically activated, and so cannot be used in in vitro models as a control. Mouse-to-mouse transmission models exist [145], but use murine parasites, and have not been sufficiently cross-validated with clinical data to allow their use in decision making. Currently, direct comparison of transmission-blocking activity can only be made from data in human subjects.

Transmission can be prevented if mature stage V gametocytes are killed or rendered non-functional, and if the development of new gametocytes can be stopped. Over the last 4 years, the ability to generate enough gametocytes to allow primary screening of large compound collections has been realized [108, 146–148]. Full characterization of compounds across a range of in vitro activities, such as exflagellation (male gamete formation), female gamete formation or oocyst inhibition provides additional insight. Compounds may also have effects on *Plasmodium* stages inside the insect vector, and these effects are currently captured under TCP-5. In the event that new compound series are found with well-defined activities on stages in the insect (clearly distinct from the endectocide activity in TCP-6 below), this will be revisited. Confirmation of the effect on transmission reduction is measured with the standard membrane feeding assay (SMFA) that measures both the number of oocysts per midgut (intensity of infection) and the number of mosquitoes infected (prevalence of infection). Considerable improvements in this assay have been made in the last 4 years [149, 150], and its outcome is now part of the standard decision-making process. Accurate efficacy prediction of the SMFA results from the gametocyte counts is currently not possible, so the recommended strategy is to use the gametocyte data as a filter to select compounds that impact transmission, but to make decisions based on SMFA data at the lead-to-candidate stage.

The link between the SMFA data and human transmission-blocking capability has also yet to be elucidated. The observation that in the CHMI model, piperaquine treatment causes a consistent decrease in the female gametocyte marker Pfs25, opens up new possibilities to use this to rank clinical candidates for their transmission-blocking activity [122]. However, a male stage V gametocyte marker is similarly required to properly interpret the potential of a compound, particularly since, from in vitro data, the majority of mechanisms appear to predominantly impact only male gametocytes [151]. Understanding the duration of exposure that is required will also be a critical factor here; artemether incapacitates gametocytes in culture [152], and an analysis of 62 studies concluded that artemether–lumefantrine has consistent gametocytocidal effects [153–155].

The clinical development pathway for an exclusively transmission-blocking compound is still not well defined. Until now, all transmission-blocking candidates have also had activity against the asexual blood stages. Discussions about the approval pathway for a transmission-blocking vaccine [156] suggest a route forwards, which could be based on finding activity in CHMIs followed by a demonstration of the reduction of infections at a community level over a prolonged period [157], then seeking approval through a standard treatment regulatory path for adding such an agent to a double combination. A strategy focused on targeting vector-stage parasites only would rely on mosquitoes biting while drug levels are still in the efficacious range. Since, in the absence of a gametocytocidal agent, infective stage V gametocytes can circulate for beyond 20 days, such a strategy is only feasible if drug concentrations are extremely protracted; this is a considerable challenge that conceivably could be overcome with intramuscular dosing or orally, with the use of particularly stable compounds. The latter is discussed in the next section in the context of medicines that kill mosquitoes following the drug’s uptake in a bloodmeal: endectocides.

## Direct effect on the insect vector (potential TCP-6)

Studies with the endectocide ivermectin, used to treat onchocerciasis (river blindness), lymphatic filariasis and strongyloidiasis, have demonstrated a significant effect on these parasites as well as the morbidity and mortality of insects feeding on subjects with plasma concentrations of the drug. Merely shortening the insect vector’s lifespan is predicted to have a significant impact on malaria transmission, since oocyte maturation is a slow, highly temperature-dependent process that requires almost all of the adult mosquito’s remaining lifetime. Widespread treatment of populations with such endectocides has been proposed as a potential approach in the eradication of malaria [158]. This TCP has been the discussion of a 2016 expert review by the WHO, and several topics are worth underlining. The optimal ivermectin regimen [159] requires a higher dose or longer duration of therapy than the single 200 μg/kg used in onchocerciasis, and for this, additional clinical safety data would be needed. Modelling is also needed to predict the human population coverage required for a significant effect, since the current label precludes women of child-bearing potential, in the absence of data on first-trimester pregnancy use. Pricing is unlikely to be an issue; ivermectin is relatively cheap, at $750/kg, and the doses are low. Ultimately, the challenge is that the half-life of ivermectin in humans is 18–35 h, suggesting that it is likely to have a short duration of action. Long-acting formulations have been proposed for such a TCP, and also newer generations of veterinary-approved isoxazolines have 3 months of coverage, and offer an alternative route forward.

## Portfolio considerations for choosing combination components

One of the most difficult issues with managing the portfolio is navigating the maze between the early studies (up to clinical proof of concept in volunteers or patients) and the later stages, where compounds are used exclusively in combination. Selection of which molecules to combine, and how, depends principally on four factors: efficacy, safety, propensity to generate resistance, and ability to be co-formulated:

### Efficacy

For efficacy, simple isobolograms are often used to identify highly significant synergies in the partners’ IC_50_s, and a lack of significant antagonism. However, these rely on parasite growth inhibition assays, which give no information on ‘killing’; the most relevant in vitro studies involve examining the effect of combinations (at different concentrations of each drug) in the PRR assay, since this informs with respect to synergistic, additive or antagonistic effects on the rate of parasite killing. This is the closest in vitro endpoint to mirror what is then measured in mice and humans: in vivo parasite clearance. The next stage will be to better understand whether any interaction is seen between two compounds when they are dosed as a combination in the in vivo NOD SCID mouse model, where the speed of killing is assessed, but also the initial delay in onset of activity. These results could then be used to guide testing of combinations in the CHMI model in volunteers. Critically, both the in vitro and in vivo mouse and human data can be modelled to investigate alterations in the potency (MIC and MPC), the PRR and time to recrudesce. Combined with modelling, this should provide a clear rationale for partner and dose range selection in the phase IIb studies. In the event that single doses of two molecules do not provide sufficient coverage to achieve cures in at least 95% of the patients, then the next approach would be to extend to three or more molecules. This is not new for malaria either: artesunate–sulfadoxine–pyrimethamine is already widely used in India. However, the triple combination chlorproguanil–dapsone–artesunate failed for safety issues related to dapsone; moreover, the combination of the three active drugs did not achieve a 95% cure rate [160, 161]. Note that this triplet included molecules that each had a short half-life. Adding more drugs may generate more problems than it solves in some situations.

### Safety

For safety, there are two aspects. First, to understand the individual compound liabilities, and second to use clinical observation and metabolic modelling to establish whether two compounds have a drug interaction such that one compound inappropriately modulates the PK curve of the other, perhaps causing an increased safety risk. Such modelling typically involves physiologically based PK models, and MMV is working with Certara using the Simcyp simulator to better manage this risk [162].

### Resistance

There should be no evidence of clinically relevant resistance to either molecule. For new molecules where the resistance marker is known, an interrogation of the genomes of sequenced parasites is prudent in confirming the absence of deleterious pre-existing mutations. Furthermore, cross-resistance studies can be conducted in which each drug is tested to confirm that it can kill all resistant clones when combined with the partner drug. A combination in vitro selection study should similarly confirm a reduction in the risk of resistance generation. The ideal situation is that the compounds are individually resistance-proof (at a level of detection of 1 in 10^9^) coming from chemical series where it is difficult to raise resistance in vitro. The fitness costs for resistance may also play a role; it appears that *Plasmodium* appears unable to maintain resistance against certain pairs of drugs, such as mefloquine and piperaquine, at the same time [163]. Finding other such mutually exclusive pairings in the portfolio will be important over the next period. An emerging area of parasitological understanding is the confirmation and relevance of asexual blood-stage dormancy or quiescence, and whether certain compounds *promote* dormancy or, in contrast, can kill or re-activate any putative dormant forms [164–166]. Such resistance mechanisms are extremely complicated and more basic science is required to understand the clinical significance of in vitro studies in this area. Naturally, as the state of the art develops, the drug combination criteria develop too. If clinical dormancy is confirmed and robust assays become available, clearance of such parasites, within a combination, will be critical. Triple combinations have been mentioned above in relation to efficacy but could equally be considered from a resistance perspective to preserve the longevity of a combination. If pre-existing mutations resistant to any one drug in a double combination exist then it could be argued that the ‘double’ combination is actually ‘monotherapy’. Triple combinations would mitigate this risk.

### Formulation

Finally, the formulation compatibility question becomes important when assessing a single-dose cure. The total amount of drug used is likely to be high, considering the current molecules in the portfolio, where very few have a predicted or confirmed therapeutic dose of <100 mg. The requirement for complex formulations can therefore increase the total amount of material administered, and above certain levels, this is unacceptable. The use of complex formulations will have an impact on costs. The current formulations of ACT are priced between $1.00 and $3.00, when an adult course of therapy is purchased in a public health setting. While current therapies retain efficacy in Africa it is hard to see that new drugs with higher prices would have a significant impact on the market. If current ACT fails in Africa the value of new drugs increases dramatically, and so the minimal acceptable cost profiles will change.

## How strong is the current portfolio?

An earlier analysis [4] showed that the attrition rate in malaria medicine development closely follows the Centre for Medicines Research (CMR) international benchmarks for anti-infective medicines in general. This means that malaria drug discovery has an attrition rate that is no better and no worse than that in the pharmaceutical industry for anti-infectives overall, and significantly better than for other therapeutic areas, such as neurology and oncology. One difficulty with such an analysis is that new chemotypes have an inherently higher risk than additional members of established chemical classes, other than the risks of failure due to resistance (which are higher for drugs that have been used for many years). For this review’s new analysis (Table 8), new chemotypes are separated from life-cycle management projects. The overall success rates have not changed significantly in the last 3 years, either for MMV or for the CMR benchmark, which is reassuring. The data shows that a new molecule in formal preclinical evaluation has an 8% chance of becoming part of a product.

**Table 8:** Success rates (%) in development 2009–2014 for MMV compared to benchmark data, by phase

	MMV				CMR^a^		PBF^b^	
	Excluding LCM^c^		Including LCM					
	Per phase	Cumu-lative	Per phase	Cumu-lative	Per phase	Cumu-lative	Per phase	Cumu-lative
Preclinical	50	8	50	14	60	5	40	3
Phase I	70	16	70	27	56	9	54	7
Phase IIa	75	23	78	39	36	16	34	13
Phase IIb	60^d^	30	75	50	60^d^	45	60^d^	38
Phase III	50	50	67	67	84	75	70	64
Registration	100	100	100	100	89	89	91	91

^a^LCM: Life cycle management; these are the medicines that were brought into the MMV portfolio when it was already clear that they are well tolerated and effective, but the task was to generate new formulations or co-formulations

^b^Pharmaceutical Benchmarking Forum; CMR data 2013

^c^PBF data 2010

^d^Stage success rate of 60% for combining two medicines has been added into reflect the potential for unfavourable drug–drug interactions that prevents further development of a combination. However, as discussed in the text, this may be an underestimate, since it does not include additional risk because of the change in endpoints between parasite reduction in phase IIa (APCR on day 14 or 28) and ACPR day 28 in phase IIb. No additional allowance has been made for the risk that a medicine may fail because it is not possible to produce a pediatric presentation

The naïve interpretation of this is that no more than 13 candidate compounds would be needed for a reasonable chance of launching one new medicine, and 25 for a combination with two NCEs (new chemical entities). However, if one aims for a 90% overall chance of success (P), the number of candidates n, each with a success probability s (8%) is described by the negative binomial distribution in Eq. 1.

1 $$ \left( {1 - P} \right) = (1 - s)^{n} + \left[ {\left( {1 - s} \right)^{n - 1} *s} \right]*\left( {\begin{array}{*{20}c} n \\ 1 \\ \end{array} } \right) $$

This equation represents the relationship between P, or the overall probability of discovering two or more successful medicines, the number n of candidate molecules pursued, and success probability s for each candidate.

In this expression the left term denotes the overall *failure* to discover at least two successful medicines from a set of n candidate molecules. The terms on the right add the probabilities of finding zero, or exactly one successful medicine when following up n candidate molecules. The numerical solutions for this equation show that at least 48 molecules are to be evaluated for this probability to exceed 90%. The MMV discovery portfolio was built and funded to deliver two new preclinical candidates per year, and allowing for a third molecule coming from elsewhere, then this would still require some 16 years’ investment per combination, except if there is a considerable increase in success rates going forwards (and a justification for this), or significantly increased investment. The hope on the horizon is that many of the new molecules are first-in-class, and so should they fail in clinical development there will be scope for well-defined back-up projects, and these are normally much more cost-effective programmes. The definition of new molecular targets also opens up new possibilities for engaging the wider research community, allowing access to resources such as the EU’s European Lead Factory, which considers molecular targets but not phenotypic approaches.

**Fig. 3:**
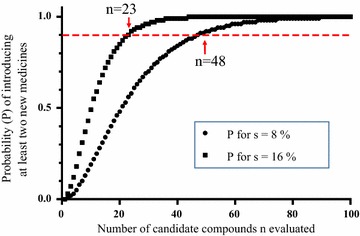
Probability (P) of delivering two or more new medicines after evaluating n candidate molecules, each with a probability of success (s) of 8 or 16%. The *red line* indicates an overall delivery success of 90%, with minimal n-values indicated for each curve

The factors underlying these metrics merit closer attention. The increased stringency of review of projects at the lead and preclinical candidate stages means that the quality of compounds is theoretically improving, so future success rates may be underestimated [112]. Of the 16 molecules entering preclinical development from the MMV portfolio since 2009, only four have been abandoned, with six moving successfully to phase I, and a further seven working their way through safety studies, with four new phase I starts expected in 2017. As Fig. 3 with plots for Eq. 1 shows, doubling the success rate for individual candidate molecules (from 8 to 16%) more than halves the number that need to be evaluated. One area for possible improvement is the success rate in phase III, which is currently only 50%. The frequent phase III failures in other therapeutic areas have been linked to premature decisions to proceed to phase III, due to external, non-scientific pressures, without appropriately strong phase II data [167]. On the other hand it is difficult to estimate the additional risk factor for putting together new compounds in phase IIb, cited as 60%. However, this is still only an estimate.

It also remains a question as to whether this level of delivery of preclinical candidates can be maintained. The initial momentum was provided by phenotypic screening of diverse compound collections. New chemical diversity has been screened in the last 4 years, including microbial metabolites from Japan, and molecules with novel chemistries such as boron [168, 169] and increased sp3 types [170]. These sources are rapidly being mined out and it is only through access to novel chemical diversity via new pharmaceutical compound libraries that new relevant compounds can be screened. Examples for these creative sources include compounds that populate Lilly’s Open Innovation Drug Discovery platform [171] or the Community for Open Antimicrobial Drug Discovery (COADD) [172] and the direct selection of compounds for screening that come from emerging in silico models [173]. Other strategies include investigating compounds in the 1–10 μM range of potency (which up until now have been considered too weak) and retesting those compounds already screened in new assays, not least phenotypic assays, against non-asexual blood stages of the parasite. An exciting development is the emergence of new, validated molecular targets [6], which have the advantage that they are already known to be druggable, often with in vivo, and sometimes clinical validation. Some of these targets are enzymes: DHODH, PI-4 kinase and the elongation factor EF2, where the three-dimensional structures may be accessible, driving target-based discovery of new scaffolds. In the event of increased attrition in the early clinical pipeline, there will be a potential for fast-follower, next-generation projects to fix the issues; these must be ‘differentiated back-ups’, rather than simple ‘me-too’ compounds. The next-generation compound will be specifically designed to overcome the problems encountered by the first compounds. Testing multiple, diverse scaffolds against the same pathway is the only way to ensure ultimate delivery.

## Discussion

The malaria eradication agenda has set new challenges for drug discovery. The central hypothesis is that controlling malaria will always be a costly activity, requiring a continual pipeline of new medicines to overcome acquired drug resistance as and when it emerges. Most elimination strategies proposed require an initial reduction in transmission by vector control, followed by multiple rounds of medicines used in MST or MDA settings, and finally methods to stop re-introduction, or to prevent epidemics. New medicines will need exquisite levels of clinical safety and tolerability. On top of this, the new medicines need to break the transmission cycle. Although there has been considerable success over the last 4 years in finding molecules with transmission-blocking potential, it is only now that early discovery is able to start with that end in mind, and screen primary diversity for this potential rather than retro-fitting it onto blood-stage actives.

Against these exceptionally high hurdles, there is a much stronger portfolio of new preclinical candidates. Most of the newer compounds were selected because of a significant PRR, at least in vitro, and were optimized to maximize their plasma residence time. The new generation of compounds kills as fast as mefloquine, or faster, and can maintain activity for up to 8 days, and the risk of unmatched pharmacological duration of cover is somewhat diminished [115]. This review has simplified the classification, describing all compounds with activity against blood stages as TCP-1, provided that they are predicted to be capable of a reduction in parasitemia of at least 10^6^-fold in humans. But ideally a new molecule should be able to kill all the parasites in all patient groups, with a 10^12^-fold reduction of parasitemia at exposures equivalent to those in the lowest 5% of the population. In reality one can only measure 10^6^–10^7^-fold drops in parasitemia by PCR, and so any number greater than this is an extrapolation.

Making sure that compounds progress quickly through the pipeline is important. Preclinical development can be achieved at ‘industrial speeds’, as illustrated by the 14 months needed for artefenomel. However the cost in clinical development rises steeply from phase I to phase III. Activities can be carried out in parallel, however the cost/risk impact needs to be carefully considered, and streamlined decision-making processes and full up-front funding are required. The availability of human challenge models has accelerated development for two reasons. First, it gives increased confidence in the molecule and this is important in allowing timely decisions to invest in the later clinical phases. Second, the selection of doses for phase II can be informed early in phase I, allowing early submission of clinical protocols before phase I is complete. This can significantly accelerate initiation of phase II studies, since the approval of these protocols in disease-endemic countries can take between 4 and 18 months. Early confidence in molecules is a critical success factor.

For anti-relapse molecules, there has been progress over the last 4 years, but no new candidates yet. Tafenoquine has demonstrated anti-relapse efficacy at a single dose of 300 mg, and is now on the cusp of regulatory submission. However, there are no new molecules with TCP-3 activity following it in the pipeline. The major development is that there are now at last cellular assays capable of testing hundreds of molecules for this TCP, and this may spur a new era in anti-relapse drug discovery over the next few years, although there is no certainty.

The pipeline has been significantly enriched in new molecules with activity against hepatic schizonts, which could be used in chemoprotection and perhaps chemoprevention regimens. Around half of the chemotypes identified by their activity against asexual stages have activity against hepatic schizonts. These include KAF156 [9, 174], DSM265 [11], MMV048 and DDD498 [6].

Finally, there are a large number of new molecules which display activity in transmission-blocking assays in vitro, at concentrations that might be achieved clinically. Examples already in clinical development include OZ439, KAE609, KAF156, SJ733, and DDD498. The ability to test for activity against gametocyte survival over time, and against viable transmission to insects in CHMIs will help a better understanding of the combination of cellular activities and clinical PK required to achieve a clinical effect. Ideally one of the two (or three) drugs in a new combination would have transmission-blocking activity. An alternative approach would be to add a low dose of primaquine to a combination, provided that primaquine does not have a negative drug–drug interaction with one of the blood schizonticides.

## Conclusions

The shift from control to elimination of malaria set ambitious goals for treatment and chemoprevention of malaria, at a time when only a handful of new molecules entered preclinical development over the preceding decade. The last 4 years has seen the continued arrival of new molecules, a deepening understanding of the molecules in the pipeline, a more profound understanding of how to combine these molecules, and, of course, lessons learned from attrition in the pipeline. The use of early clinical studies allowing for longer term follow-up (to 28 days) or controlled human malaria infection studies allows characterization of the key performance parameters such as the PRR and the MIC and MPC for each molecule, and in combination.

Over the next 4-year period an understanding of how to translate these parameters into the key output for new treatments (ACPR and cure) will deepen as a result of having more clinical phase IIb data. These should provide a more quantitative framework for assessing the impact of patient genetic background and immune status. Resistance continues to be an issue; all of the new medicines must be as resistance proof as possible, and discussion about the medicines needed in severe malaria should artemisinin fail needs to be revived. The largest gap in the portfolio is the absence of molecules for the anti-relapse profiles, although there is some optimism now that cellular assays start to become available. For transmission blocking, there are now many molecules with good in vitro activity, and over the next few years these will be tested in CHMI models to see how their transmission blocking activity compares to low-dose primaquine. New endectocide approaches have been proposed, but are still at an early stage, and their ultimate hurdle will clearly be the adoption by malaria-affected countries. There are also several molecules with hepatic schizont activity, with potential for use in chemoprotection, and sporozoite challenge models will provide data to help refine predictions on how to link cellular activity and human PK to better predict clinical activity. Such a medicine is vital in the final phases of eradication where maintaining zero transmission during potential epidemics is critical. The current chemoprotection options are extremely limited: atovaquone-proguanil, because of its daily administration, and mefloquine because of its safety profile.

The recently published Global Technical Strategy for Malaria sets out an aggressive agenda for reducing malaria incidents and deaths by 90% over the period 2016–2030 [3]. This plan is aggressive in that such a fall is more significant than that seen over the previous 15 years, and is set against a continuing threat that multidrug-resistant malaria may emerge in Africa at any point during that period. However, even allowing for success, there would still be an unacceptable 40,000 deaths and 20 million cases of malaria per year, and a need for new medicines to be launched in the decade after this timeframe. To continue the current rate of productivity will require new strategies for hunting out and designing clinical candidates, and continued increases in efficiency to not only test compounds, but pick the winners early. In short, this will require a continual focus on finding the transformative new medicines described by these target product profiles.

## Abbreviations

(S)AE: (Serious) adverse event
ACPR: adequate clinical and parasitological response (at day 28)
ACT: artemisinin combination therapy
AIDS: acquired immune deficiency syndrome
ATQ: atovaquone
AUC: area under the curve
CHMI: controlled human malaria infection
CMR: Centre for Medicines Research
DHA: dihydroartemisinin
DHFR: dihydrofolate reductase
DHODH: dihydroorotate dehydrogenase
DMPK: drug metabolism and PK
EC/D90: concentration/dose that results in 90% suppression of parasitemia
ECG: electrocardiogram
EFD: embryo-fetal development
EMA: European Medicines Agency
FDA: Food and Drug Administration
G6PD: glucose-6-phosphate dehydrogenase
GMS: greater Mekong sub-region
HIV: human immunodeficiency virus
ICH: International council on harmonization
IPTi: intermittent preventive treatment in infants
IPTp: intermittent preventive treatment in pregnancy
malERA: malaria eradication agenda
MDA: mass drug administration
MIC: minimum inhibitory concentration
MMV: medicines for malaria venture
MPC: minimum parasiticidal concentration
MRI: magnetic resonance imaging
MST: mass screen-and-treat
NOAEL: no observed adverse effect level (dose)
NOD SCID: non-obese diabetes, severe immunodeficient
PI4K: phosphatidylinosine-4-kinase
PCR: polymerase chain reaction
PD: pharmacodynamic(s)
PK: pharmacokinetic(s)
PrEP: pre-exposure prophylaxis
PRR: parasite reduction rate
RDT: rapid diagnostic test
RH: relative humidity
SDC: single dose cure
SEC: single exposure chemoprotection
SERC: single encounter radical cure
SERCaP: single encounter radical cure and post-treatment prophylaxis
SMC: seasonal malaria chemoprevention
SMFA: standard membrane feeding assay
SRA: stringent regulatory agency
TB: tuberculosis
TCP: target candidate profile
TPP: target product profile
WHO: World Health Organization

## References

[1] WHO. World Malaria Report 2015. Geneva, World Health Organization, 2015. http://www.whoint/malaria/publications/world-malaria-report-2015/report/en/. 2015.

[2] malERA Consultative Group on Drugs (2011). A research agenda for malaria eradication: drugs. PLoS Med..

[3] WHO. Global Technical Strategy for Malaria 2016–2030. Geneva, World Health Organization. http://www.whoint/malaria/areas/global_technical_strategy/en/. 2016.

[4] Burrows JN, Hooft van Huijsduijnen R, Möhrle JJ, Oeuvray C, Wells TNC (2013). Designing the next generation of medicines for malaria control and eradication. Malar J..

[5] USFDA. Guidance for industry and review staff target product profile—a strategic development process tool. Draft guidance. http://www.fdagov/downloads/drugs/guidancecomplianceregulatoryinformation/guidances/ucm080593pdf. 2007.

[6] Wells TNC, Hooft van Huijsduijnen R, Van Voorhis WC (2015). Malaria medicines: a glass half full?. Nat Rev Drug Discov..

[7] Phyo AP, Jittamala P, Nosten FH, Pukrittayakamee S, Imwong M, White NJ (2016). Antimalarial activity of artefenomel (OZ439), a novel synthetic antimalarial endoperoxide, in patients with *Plasmodium falciparum* and *Plasmodium vivax* malaria: an open-label phase 2 trial. Lancet Infect Dis..

[8] White NJ, Pukrittayakamee S, Phyo AP, Rueangweerayut R, Nosten F, Jittamala P (2014). Spiroindolone KAE609 for falciparum and vivax malaria. N Engl J Med.

[9] Leong FJ, Zhao R, Zeng S, Magnusson B, Diagana TT, Pertel P (2014). A first-in-human randomized, double-blind, placebo-controlled, single- and multiple-ascending oral dose study of novel Imidazolopiperazine KAF156 to assess its safety, tolerability, and pharmacokinetics in healthy adult volunteers. Antimicrob Agents Chemother.

[10] White NJ, Duong TT, Uthaisin C, Nosten F, Phyo AP, Hanboonkunupakarn B (2016). Antimalarial activity of KAF156 in falciparum and vivax malaria. N Engl J Med.

[11] Phillips MA, Lotharius J, Marsh K, White J, Dayan A, White KL (2015). A long-duration dihydroorotate dehydrogenase inhibitor (DSM265) for prevention and treatment of malaria. Sci Transl Med..

[12] Ariey F, Witkowski B, Amaratunga C, Beghain J, Langlois AC, Khim N (2014). A molecular marker of artemisinin-resistant *Plasmodium falciparum* malaria. Nature.

[13] Amaratunga C, Lim P, Suon S, Sreng S, Mao S, Sopha C (2016). Dihydroartemisinin–piperaquine resistance in *Plasmodium falciparum* malaria in Cambodia: a multisite prospective cohort study. Lancet Infect Dis..

[14] Leang R, Taylor WR, Bouth DM, Song L, Tarning J, Char MC (2015). Evidence of *Plasmodium falciparum* malaria multidrug resistance to artemisinin and piperaquine in Western Cambodia: dihydroartemisinin–piperaquine open-label multicenter clinical assessment. Antimicrob Agents Chemother.

[15] Tacoli C, Gai PP, Bayingana C, Sifft K, Geus D, Ndoli J (2016). Artemisinin resistance-associated K13 polymorphisms of *Plasmodium falciparum* in Southern Rwanda, 2010–2015. Am J Trop Med Hyg.

[16] Chen I, Clarke SE, Gosling R, Hamainza B, Killeen G, Magill A (2016). “Asymptomatic” malaria: a chronic and debilitating infection that should be treated. PLoS Med..

[17] WHO. Single dose Primaquine as a gametocytocide in *Plasmodium falciparum* malaria: updated WHO policy recommendation (2012). http://www.whoint/malaria/pq_updated_policy_recommendation_en_102012pdf. 2012.

[18] Chaccour C, Killeen GF (2016). Mind the gap: residual malaria transmission, veterinary endectocides and livestock as targets for malaria vector control. Malar J..

[19] http://www.endmalaria2040.org.

[20] Ndiaye JL, Randrianarivelojosia M, Sagara I, Brasseur P, Ndiaye I, Faye B (2009). Randomized, multicentre assessment of the efficacy and safety of ASAQ—a fixed-dose artesunate–amodiaquine combination therapy in the treatment of uncomplicated *Plasmodium falciparum* malaria. Malar J..

[21] Sagara I, Beavogui AH, Zongo I, Soulama I, Borghini-Fuhrer I, Fofana B (2016). Safety and efficacy of re-treatments with pyronaridine–artesunate in African patients with malaria: a substudy of the WANECAM randomised trial. Lancet Infect Dis..

[22] Khatib RA, Selemani M, Mrisho GA, Masanja IM, Amuri M, Njozi MH (2013). Access to artemisinin-based anti-malarial treatment and its related factors in rural Tanzania. Malar J..

[23] Hastings IM, Hodel EM, Kay K (2016). Quantifying the pharmacology of antimalarial drug combination therapy. Sci Rep..

[24] Valecha N, Looareesuwan S, Martensson A, Abdulla SM, Krudsood S, Tangpukdee N (2010). Arterolane, a new synthetic trioxolane for treatment of uncomplicated *Plasmodium falciparum* malaria: a phase II, multicenter, randomized, dose-finding clinical trial. Clin Infect Dis.

[25] Stein DS, Jain JP, Kangas M, Lefevre G, Machineni S, Griffin P (2015). Open-label, single-dose, parallel-group study in healthy volunteers to determine the drug-drug interaction potential between KAE609 (Cipargamin) and piperaquine. Antimicrob Agents Chemother.

[26] McCarthy JS, Baker M, O’Rourke P, Marquart L, Griffin P, Hooft van Huijsduijnen R (2016). Efficacy of OZ439 (artefenomel) against early *Plasmodium falciparum* blood-stage malaria infection in healthy volunteers. J Antimicrob Chemother.

[27] McCarthy JS, Lotharius J, Dayan A, Phillips M, Marsh K, Walker D (2014). A phase I/Ib study to investigate the safety, tolerability and pharmacokinetic profile of DSM265 in healthy subjects and then its antimalarial activity in induced blood stage *Plasmodium falciparum* infection. ASTMH Ann. Meet..

[28] Rückle T, Duparc S, Kerr N, Rosario M, Qiu P, Phillips MA, et al. A phase IIa proof-of-concept study to assess the efficacy, safety, tolerability and pharmacokinetics of single doses of DSM265 in adult patients with acute, uncomplicated *Plasmodium falciparum* or *vivax* malaria mono-infection over a 28-day-extended observation period in Iquitos, Peru. In: ASTMH Annual Meeting. 2014;857. Philadelphia, USA, 2015.

[29] Held J, Supan C, Salazar CL, Tinto H, Bonkian LN, Nahum A (2015). Ferroquine and artesunate in African adults and children with *Plasmodium falciparum* malaria: a phase 2, multicentre, randomised, double-blind, dose-ranging, non-inferiority study. Lancet Infect Dis..

[30] McCarthy JS, Marquart L, Sekuloski S, Trenholme K, Elliott S, Griffin P (2016). Linking murine and human *Plasmodium falciparum* challenge models in a translational path for antimalarial drug development. Antimicrob Agents Chemother.

[31] Zani B, Gathu M, Donegan S, Olliaro PL, Sinclair D (2014). Dihydroartemisinin–piperaquine for treating uncomplicated *Plasmodium falciparum* malaria. Cochrane Database Syst Rev..

[32] Baiden R, Oduro A, Halidou T, Gyapong M, Sie A, Macete E (2015). Prospective observational study to evaluate the clinical safety of the fixed-dose artemisinin-based combination Eurartesim(R) (dihydroartemisinin/piperaquine), in public health facilities in Burkina Faso, Mozambique, Ghana, and Tanzania. Malar J..

[33] Duparc S, Borghini-Fuhrer I, Craft CJ, Arbe-Barnes S, Miller RM, Shin CS (2013). Safety and efficacy of pyronaridine–artesunate in uncomplicated acute malaria: an integrated analysis of individual patient data from six randomized clinical trials. Malar J..

[34] Griffin JB, Lokomba V, Landis SH, Thorp JM, Herring AH, Tshefu AK (2012). *Plasmodium falciparum* parasitaemia in the first half of pregnancy, uterine and umbilical artery blood flow, and foetal growth: a longitudinal Doppler ultrasound study. Malar J..

[35] Nambozi M, Van Geertruyden JP, Hachizovu S, Chaponda M, Mukwamataba D, Mulenga M (2011). Safety and efficacy of dihydroartemisinin–piperaquine versus artemether–lumefantrine in the treatment of uncomplicated *Plasmodium falciparum* malaria in Zambian children. Malar J..

[36] EHO. Malaria in pregnancy. WHO Evidence Review Group meeting report. Geneva, World Health Organization. http://www.who.int/malaria/mpac/mpac-sept2015-erg-mip-report.pdf. 2015.

[37] Manyando C, Mkandawire R, Puma L, Sinkala M, Mpabalwani E, Njunju E (2010). Safety of artemether–lumefantrine in pregnant women with malaria: results of a prospective cohort study in Zambia. Malar J..

[38] Moore BR, Salman S, Davis TM (2016). Treatment regimens for pregnant women with falciparum malaria. Expert Rev Anti Infect Ther..

[39] Mosha D, Mazuguni F, Mrema S, Sevene E, Abdulla S, Genton B (2014). Safety of artemether–lumefantrine exposure in first trimester of pregnancy: an observational cohort. Malar J..

[40] Piola P, Nabasumba C, Turyakira E, Dhorda M, Lindegardh N, Nyehangane D (2010). Efficacy and safety of artemether–lumefantrine compared with quinine in pregnant women with uncomplicated *Plasmodium falciparum* malaria: an open-label, randomised, non-inferiority trial. Lancet Infect Dis..

[41] Spangenberg T, Burrows JN, Kowalczyk P, McDonald S, Wells TN, Willis P (2013). The open access malaria box: a drug discovery catalyst for neglected diseases. PLoS ONE.

[42] Corey VC, Lukens AK, Istvan ES, Lee MC, Franco V, Magistrado P (2016). A broad analysis of resistance development in the malaria parasite. Nat Commun..

[43] McCarthy JS, Rückle T, Djeriou E, Cantalloube C, Ter-Minassian D, Baker M (2016). A Phase II pilot trial to evaluate safety and efficacy of ferroquine against early *Plasmodium falciparum* in an induced blood-stage malaria infection study. Malar J..

[44] Sawa P, Shekalaghe SA, Drakeley CJ, Sutherland CJ, Mweresa CK, Baidjoe AY (2013). Malaria transmission after artemether–lumefantrine and dihydroartemisinin–piperaquine: a randomized trial. J Infect Dis.

[45] Daher W, Biot C, Fandeur T, Jouin H, Pelinski L, Viscogliosi E (2006). Assessment of *Plasmodium falciparum* resistance to ferroquine (SSR97193) in field isolates and in W2 strain under pressure. Malar J..

[46] Hamed K, Kuhen K (2015). No robust evidence of lumefantrine resistance. Antimicrob Agents Chemother.

[47] Beck HP, Wampfler R, Carter N, Koh G, Osorio L, Rueangweerayut R (2016). Estimation of tafenoquine anti-relapse efficacy using *Plasmodium vivax* genotyping. J Infect Dis.

[48] Rochford R, Ohrt C, Baresel PC, Campo B, Sampath A, Magill AJ (2013). Humanized mouse model of glucose 6-phosphate dehydrogenase deficiency for in vivo assessment of hemolytic toxicity. Proc Natl Acad Sci USA.

[49] Wallis RS, Maeurer M, Mwaba P, Chakaya J, Rustomjee R, Migliori GB (2016). Tuberculosis-advances in development of new drugs, treatment regimens, host-directed therapies, and biomarkers. Lancet Infect Dis..

[50] Bhatti AB, Usman M, Kandi V (2016). Current scenario of HIV/AIDS, treatment options, and major challenges with compliance to antiretroviral therapy. Cureus..

[51] Du Pont-Thibodeau G, Joyal JS, Lacroix J (2014). Management of neonatal sepsis in term newborns. F1000Prime Rep..

[52] Kateera F, Ingabire CM, Hakizimana E, Kalinda P, Mens PF, Grobusch MP (2015). Malaria, anaemia and under-nutrition: three frequently co-existing conditions among preschool children in rural Rwanda. Malar J..

[53] Rosenke K, Adjemian J, Munster VJ, Marzi A, Falzarano D, Onyango CO (2016). Plasmodium parasitemia associated with increased survival in Ebola virus-infected patients. Clin Infect Dis.

[54] Laishram DD, Sutton PL, Nanda N, Sharma VL, Sobti RC, Carlton JM (2012). The complexities of malaria disease manifestations with a focus on asymptomatic malaria. Malar J..

[55] Kimani J, Phiri K, Kamiza S, Duparc S, Ayoub A, Rojo R (2016). Efficacy and safety of azithromycin-chloroquine versus sulfadoxine-pyrimethamine for intermittent preventive treatment of *Plasmodium falciparum* malaria infection in pregnant women in Africa: an open-label, randomized trial. PLoS ONE.

[56] Malaria Hemingway J (2015). Fifteen years of interventions. Nature.

[57] Penny MA, Verity R, Bever CA, Sauboin C, Galactionova K, Flasche S (2015). Public health impact and cost-effectiveness of the RTS, S/AS01 malaria vaccine: a systematic comparison of predictions from four mathematical models. Lancet.

[58] Rts S (2015). Clinical Trials Partnership. Efficacy and safety of RTS, S/AS01 malaria vaccine with or without a booster dose in infants and children in Africa: final results of a phase 3, individually randomised, controlled trial. Lancet.

[59] Epstein JE, Richie TL (2013). The whole parasite, pre-erythrocytic stage approach to malaria vaccine development: a review. Curr Opin Infect Dis..

[60] Gomez-Perez GP, Legarda A, Munoz J, Sim BK, Ballester MR, Dobano C (2015). Controlled human malaria infection by intramuscular and direct venous inoculation of cryopreserved *Plasmodium falciparum* sporozoites in malaria-naive volunteers: effect of injection volume and dose on infectivity rates. Malar J..

[61] Lyke KE, Laurens MB, Strauss K, Adams M, Billingsley PF, James E (2015). Optimizing intradermal administration of cryopreserved *Plasmodium falciparum* sporozoites in controlled human malaria infection. Am J Trop Med Hyg.

[62] Talley AK, Healy SA, Finney OC, Murphy SC, Kublin J, Salas CJ (2014). Safety and comparability of controlled human *Plasmodium falciparum* infection by mosquito bite in malaria-naive subjects at a new facility for sporozoite challenge. PLoS ONE.

[63] Spinner CD, Boesecke C, Zink A, Jessen H, Stellbrink HJ, Rockstroh JK (2016). HIV pre-exposure prophylaxis (PrEP): a review of current knowledge of oral systemic HIV PrEP in humans. Infection.

[64] Margolis DA, Boffito M (2015). Long-acting antiviral agents for HIV treatment. Curr Opin HIV AIDS..

[65] WHO. WHO policy recommendation: Seasonal malaria chemoprevention (SMC) for *Plasmodium falciparum* malaria control in highly seasonal transmission areas of the Sahel sub-region in Africa. Geneva, World Health Organization. http://www.whoint/malaria/publications/atoz/who_smc_policy_recommendation/en/. 2012.

[66] Cairns M, Roca-Feltrer A, Garske T, Wilson AL, Diallo D, Milligan PJ (2012). Estimating the potential public health impact of seasonal malaria chemoprevention in African children. Nat Commun..

[67] Noor AM, Kibuchi E, Mitto B, Coulibaly D, Doumbo OK, Snow RW (2015). Sub-national targeting of seasonal malaria chemoprevention in the Sahelian countries of the Nouakchott Initiative. PLoS ONE.

[68] Tagbor H, Antwi GD, Acheampong PR, Bart Plange C, Chandramohan D, Cairns M (2016). Seasonal malaria chemoprevention in an area of extended seasonal transmission in Ashanti, Ghana: an individually randomised clinical trial. Trop Med Int Health..

[69] Tine RC, Ndour CT, Faye B, Cairns M, Sylla K, Ndiaye M (2014). Feasibility, safety and effectiveness of combining home based malaria management and seasonal malaria chemoprevention in children less than 10 years in Senegal: a cluster-randomised trial. Trans R Soc Trop Med Hyg.

[70] Zongo I, Milligan P, Compaore YD, Some AF, Greenwood B, Tarning J (2015). Randomized noninferiority trial of dihydroartemisinin–piperaquine compared with sulfadoxine-pyrimethamine plus amodiaquine for seasonal malaria chemoprevention in Burkina Faso. Antimicrob Agents Chemother.

[71] Matondo SI, Temba GS, Kavishe AA, Kauki JS, Kalinga A, van Zwetselaar M (2014). High levels of sulphadoxine–pyrimethamine resistance *Pfdhfr*-*Pfdhps* quintuple mutations: a cross sectional survey of six regions in Tanzania. Malar J..

[72] WHO. Guidelines for th treatment of malaria, Third edition. Geneva, World Health Organization. http://www.appswhoint/iris/bitstream/10665/162441/1/9789241549127_engpdf. 2015.

[73] Milner DA, Whitten RO, Kamiza S, Carr R, Liomba G, Dzamalala C (2014). The systemic pathology of cerebral malaria in African children. Front Cell Infect Microbiol..

[74] Dondorp AM, Fanello CI, Hendriksen IC, Gomes E, Seni A, Chhaganlal KD (2010). Artesunate versus quinine in the treatment of severe falciparum malaria in African children (AQUAMAT): an open-label, randomised trial. Lancet.

[75] Dondorp A, Nosten F, Stepniewska K, Day N, White N (2005). Artesunate versus quinine for treatment of severe falciparum malaria: a randomised trial. Lancet.

[76] Kremsner PG, Taylor T, Issifou S, Kombila M, Chimalizeni Y, Kawaza K (2012). A simplified intravenous artesunate regimen for severe malaria. J Infect Dis.

[77] Kremsner PG, Adegnika AA, Hounkpatin AB, Zinsou JF, Taylor TE, Chimalizeni Y (2016). Intramuscular srtesunate for severe malaria in African children: a multicenter randomized controlled trial. PLoS Med..

[78] Barrocas AM, Cymet T (2007). Cinchonism in a patient taking quinine for leg cramps. Compr Ther.

[79] Wolf LR, Otten EJ, Spadafora MP (1992). Cinchonism: two case reports and review of acute quinine toxicity and treatment. J Emerg Med.

[80] Centers for Disease Control (2013). Published reports of delayed hemolytic anemia after treatment with artesunate for severe malaria—worldwide, 2010–2012. MMWR Morb Mortal Wkly Rep.

[81] Jaureguiberry S, Ndour PA, Roussel C, Ader F, Safeukui I, Nguyen M (2014). Postartesunate delayed hemolysis is a predictable event related to the lifesaving effect of artemisinins. Blood.

[82] Rolling T, Agbenyega T, Krishna S, Kremsner PG, Cramer JP (2015). Delayed hemolysis after artesunate treatment of severe malaria—review of the literature and perspective. Travel Med Infect Dis..

[83] Okebe J, Eisenhut M (2014). Pre-referral rectal artesunate for severe malaria. Cochrane Database Syst Rev..

[84] Ashley EA, Dhorda M, Fairhurst RM, Amaratunga C, Lim P, Suon S (2014). Spread of artemisinin resistance in *Plasmodium falciparum* malaria. N Engl J Med.

[85] Cheah PY, Parker M, Dondorp AM (2016). Development of drugs for severe malaria in children. Int Health..

[86] Deng X, Duffy SP, Myrand-Lapierre ME, Matthews K, Santoso AT, Du YL (2015). Reduced deformability of parasitized red blood cells as a biomarker for anti-malarial drug efficacy. Malar J..

[87] Jimenez-Diaz MB, Ebert D, Salinas Y, Pradhan A, Lehane AM, Myrand-Lapierre ME (2014). (+)-SJ733, a clinical candidate for malaria that acts through ATP4 to induce rapid host-mediated clearance of Plasmodium. Proc Natl Acad Sci USA..

[88] Flannery EL, McNamara CW, Kim SW, Kato TS, Li F, Teng CH (2015). Mutations in the P-type cation-transporter ATPase 4, PfATP4, mediate resistance to both aminopyrazole and spiroindolone antimalarials. ACS Chem Biol.

[89] Spillman NJ, Kirk K (2015). The malaria parasite cation ATPase PfATP4 and its role in the mechanism of action of a new arsenal of antimalarial drugs. Int J Parasitol Drugs Drug Resist..

[90] Telen MJ, Batchvarova M, Shan S, Bovee-Geurts PH, Zennadi R, Leitgeb A (2016). Sevuparin binds to multiple adhesive ligands and reduces sickle red blood cell-induced vaso-occlusion. Br J Haematol.

[91] Higgins SJ, Elphinstone RE, Kain KC. Adjunctive Therapies for Malaria. In Living Reference Work. Volume Encyclopedia of Malaria. Edited by Kremsner PG, Krishna S. New York: Springer New York; 2014: 1-18

[92] Taylor TE, Molyneux ME (2015). The pathogenesis of pediatric cerebral malaria: eye exams, autopsies, and neuroimaging. Ann N Y Acad Sci.

[93] Gallego-Delgado J, Walther T, Rodriguez A (2016). The high blood pressure-malaria protection hypothesis. Circ Res.

[94] Dellicour S, Desai M, Aol G, Oneko M, Ouma P, Bigogo G (2015). Risks of miscarriage and inadvertent exposure to artemisinin derivatives in the first trimester of pregnancy: a prospective cohort study in western Kenya. Malar J..

[95] McGready R, Lee SJ, Wiladphaingern J, Ashley EA, Rijken MJ, Boel M (2012). Adverse effects of falciparum and vivax malaria and the safety of antimalarial treatment in early pregnancy: a population-based study. Lancet Infect Dis..

[96] Breitkreutz J, Boos J (2007). Paediatric and geriatric drug delivery. Expert Opin Drug Deliv.

[97] Baber N (1994). International conference on harmonisation of technical requirements for registration of pharmaceuticals for human use (ICH). Br J Clin Pharmacol.

[98] Shimp RL, Rowe C, Reiter K, Chen B, Nguyen V, Aebig J (2013). Development of a Pfs25-EPA malaria transmission blocking vaccine as a chemically conjugated nanoparticle. Vaccine..

[99] Lee SM, Wu CK, Plieskatt J, McAdams DH, Miura K, Ockenhouse C (2016). Assessment of Pfs25 expressed from multiple soluble expression platforms for use as transmission-blocking vaccine candidates. Malar J..

[100] Li Y, Leneghan DB, Miura K, Nikolaeva D, Brian IJ, Dicks MD (2016). Enhancing immunogenicity and transmission-blocking activity of malaria vaccines by fusing Pfs25 to IMX313 multimerization technology. Sci Rep..

[101] Kapulu MC, Da DF, Miura K, Li Y, Blagborough AM, Churcher TS (2015). Comparative assessment of transmission-blocking vaccine candidates against P*lasmodium falciparum*. Sci Rep..

[102] Campo B, Vandal O, Wesche DL, Burrows JN (2015). Killing the hypnozoite–drug discovery approaches to prevent relapse in *Plasmodium vivax*. Pathog Glob Health..

[103] Deeks SGHIV (2012). Shock and kill. Nature.

[104] Savarino A, Mai A, Norelli S, El Daker S, Valente S, Rotili D (2009). “Shock and kill” effects of class I-selective histone deacetylase inhibitors in combination with the glutathione synthesis inhibitor buthionine sulfoximine in cell line models for HIV-1 quiescence. Retrovirology..

[105] Eziefula AC, Bousema T, Yeung S, Kamya M, Owaraganise A, Gabagaya G (2014). Single dose primaquine for clearance of *Plasmodium falciparum* gametocytes in children with uncomplicated malaria in Uganda: a randomised, controlled, double-blind, dose-ranging trial. Lancet Infect Dis..

[106] Goncalves BP, Tiono AB, Ouedraogo A, Guelbeogo WM, Bradley J, Nebie I (2016). Single low dose primaquine to reduce gametocyte carriage and *Plasmodium falciparum* transmission after artemether–lumefantrine in children with asymptomatic infection: a randomised, double-blind, placebo-controlled trial. BMC Med.

[107] Bousema T, Drakeley C (2011). Epidemiology and infectivity of *Plasmodium falciparum* and *Plasmodium vivax* gametocytes in relation to malaria control and elimination. Clin Microbiol Rev.

[108] Lucantoni L, Fidock DA, Avery VM (2016). A luciferase-based, high-throughput assay for screening and profiling transmission-blocking compounds against *Plasmodium falciparum* gametocytes. Antimicrob Agents Chemother.

[109] Nilsson SK, Childs LM, Buckee C, Marti M (2015). Targeting human transmission biology for malaria elimination. PLoS Pathog.

[110] Kobylinski KC, Sylla M, Chapman PL, Sarr MD, Foy BD (2011). Ivermectin mass drug administration to humans disrupts malaria parasite transmission in Senegalese villages. Am J Trop Med Hyg.

[111] Leroy D, Campo B, Ding X, Burrows JN, Cherbuin S (2014). Defining the biology component of the drug discovery strategy for malaria eradication. Trends Parasitol.

[112] Katsuno K, Burrows JN, Duncan K, Hooft van Huijsduijnen R, Kaneko T, Kita K (2015). Hit and lead criteria in drug discovery for infectious diseases of the developing world. Nat Rev Drug Discov..

[113] McCarthy JS, Sekuloski S, Griffin P, Elliott S, Marquart L, Jörg M, et al. A Phase IIa clinical trial to characterize the pharmacokinetic–pharmacodynamic relationship of piperaquine using the induced blood stage infection model. ASTMH Ann Meet. 2014; 152. http://tinyurl.com/z9ng9df

[114] McCarthy JS, Griffin PM, Sekuloski S, Bright AT, Rockett R, Looke D (2013). Experimentally induced blood-stage Plasmodium vivax infection in healthy volunteers. J Infect Dis.

[115] White NJ (2013). Pharmacokinetic and pharmacodynamic considerations in antimalarial dose optimization. Antimicrob Agents Chemother.

[116] Sanz LM, Crespo B, De-Cozar C, Ding XC, Llergo JL, Burrows JN (2012). *P. falciparum* in vitro killing rates allow to discriminate between different antimalarial mode-of-action. PLoS ONE.

[117] Linares M, Viera S, Crespo B, Franco V, Gomez-Lorenzo MG, Jimenez-Diaz MB (2015). Identifying rapidly parasiticidal anti-malarial drugs using a simple and reliable in vitro parasite viability fast assay. Malar J..

[118] Jiménez-Díaz MB, Mulet T, Viera S, Gómez V, Garuti H, Ibáñez J (2009). Improved murine model of malaria using *Plasmodium falciparum* competent strains and non-myelodepleted NOD-SCID IL2Rγ-null mice engrafted with human erythrocytes. Antimicrob Agents Chemother.

[119] Stanisic DI, Liu XQ, De SL, Batzloff MR, Forbes T, Davis CB (2015). Development of cultured *Plasmodium falciparum* blood-stage malaria cell banks for early phase in vivo clinical trial assessment of anti-malaria drugs and vaccines. Malar J..

[120] Ndiaye JL, Faye B, Diouf AM, Kuete T, Cisse M, Seck PA (2008). Randomized, comparative study of the efficacy and safety of artesunate plus amodiaquine, administered as a single daily intake versus two daily intakes in the treatment of uncomplicated falciparum malaria. Malar J..

[121] Bakshi RP, Nenortas E, Tripathi AK, Sullivan DJ, Shapiro TA (2013). Model system to define pharmacokinetic requirements for antimalarial drug efficacy. Sci Transl Med..

[122] Pasay CJ, Rockett R, Sekuloski S, Griffin P, Marquart L, Peatey C (2016). Piperaquine monotherapy of drug-susceptible *Plasmodium falciparum* infection results in rapid clearance of parasitemia but is followed by the appearance of gametocytemia. J Infect Dis.

[123] Kofi Ekue JM, Ulrich AM, Rwabwogo-Atenyi J, Sheth UK (1983). A double-blind comparative clinical trial of mefloquine and chloroquine in symptomatic falciparum malaria. Bull World Health Organ.

[124] Leang R, Canavati SE, Khim N, Vestergaard LS, Borghini Fuhrer I, Kim S (2016). Efficacy and safety of pyronaridine–artesunate for the treatment of uncomplicated *Plasmodium falciparum* malaria in western Cambodia. Antimicrob Agents Chemother.

[125] Dembele L, Franetich JF, Lorthiois A, Gego A, Zeeman AM, Kocken CH (2014). Persistence and activation of malaria hypnozoites in long-term primary hepatocyte cultures. Nat Med.

[126] Zeeman AM, Lakshminarayana SB, van der Werff N, Klooster EJ, Voorberg-van der Wel A, Kondreddi RR (2016). PI4 kinase is a prophylactic but not radical curative target in *Plasmodium vivax*-type malaria parasites. Antimicrob Agents Chemother.

[127] Khetani SR, Bhatia SN (2008). Microscale culture of human liver cells for drug development. Nat Biotechnol.

[128] Sattabongkot J, Yimamnuaychoke N, Leelaudomlipi S, Rasameesoraj M, Jenwithisuk R, Coleman RE (2006). Establishment of a human hepatocyte line that supports in vitro development of the exo-erythrocytic stages of the malaria parasites *Plasmodium falciparum* and *P. vivax*. Am J Trop Med Hyg.

[129] Hovlid ML, Winzeler EA (2016). Phenotypic screens in antimalarial drug discovery. Trends Parasitol.

[130] Ruben A, Awe A, Hoffman SL, Sim BKL, Billingsley PF, Manoj A (2013). 157 Cryopreservation of *Plasmodium falciparum* sporozoites and Sanaria^®^ PfSPZ vaccine. Cryobiology.

[131] Singh N, Barnes SJ, Jenwithisuk R, Sattabongkot J, Adams JH (2016). A simple and efficient method for cryopreservation and recovery of viable *Plasmodium vivax* and *P. falciparum* sporozoites. Parasitol Int..

[132] March S, Ng S, Velmurugan S, Galstian A, Shan J, Logan DJ (2013). A microscale human liver platform that supports the hepatic stages of *Plasmodium falciparum* and *vivax*. Cell Host Microbe.

[133] Wells TN, Burrows JN, Baird JK (2010). Targeting the hypnozoite reservoir of *Plasmodium vivax*: the hidden obstacle to malaria elimination. Trends Parasitol.

[134] Mikolajczak SA, Vaughan AM, Kangwanrangsan N, Roobsoong W, Fishbaugher M, Yimamnuaychok N (2015). *Plasmodium vivax* liver stage development and hypnozoite persistence in human liver-chimeric mice. Cell Host Microbe.

[135] Nelwan EJ, Ekawati LL, Tjahjono B, Setiabudy R, Sutanto I, Chand K (2015). Randomized trial of primaquine hypnozoitocidal efficacy when administered with artemisinin-combined blood schizontocides for radical cure of *Plasmodium vivax* in Indonesia. BMC Med.

[136] Green JA, Patel AK, Patel BR, Hussaini A, Harrell EJ, McDonald MJ (2014). Tafenoquine at therapeutic concentrations does not prolong fridericia-corrected QT interval in healthy subjects. J Clin Pharmacol.

[137] Llanos-Cuentas A, Lacerda MV, Rueangweerayut R, Krudsood S, Gupta SK, Kochar SK (2014). Tafenoquine plus chloroquine for the treatment and relapse prevention of *Plasmodium vivax* malaria (DETECTIVE): a multicentre, double-blind, randomised, phase 2b dose-selection study. Lancet.

[138] Miller JL, Murray S, Vaughan AM, Harupa A, Sack B, Baldwin M (2013). Quantitative bioluminescent imaging of pre-erythrocytic malaria parasite infection using luciferase-expressing *Plasmodium yoelii*. PLoS ONE.

[139] Vaughan AM, Kappe SH, Ploss A, Mikolajczak SA (2012). Development of humanized mouse models to study human malaria parasite infection. Future Microbiol..

[140] Ghidelli-Disse S, Lafuente-Monasterio MJ, Waterson D, Witty M, Younis Y, Paquet T (2014). Identification of *Plasmodium* PI4 kinase as target of MMV390048 by chemoproteomics. Malar J..

[141] Fasan PO (1970). Field trial of cycloguanil pamoate in the treatment and suppression of malaria in Nigerian school children: a preliminary report. Trans R Soc Trop Med Hyg.

[142] McGregor IA, Williams K, Walker GH, Rahman AK (1966). Cycloguanil pamoate in the treatment and suppression of malaria in the Gambia, West Africa. BMJ..

[143] Wyber R, Taubert K, Marko S, Kaplan EL (2013). Benzathine penicillin G for the management of RHD: concerns about quality and access, and opportunities for intervention and improvement. Glob Heart..

[144] Leang R, Khu NH, Mukaka M, Debackere M, Tripura R, Kheang ST (2016). An optimised age-based dosing regimen for single low-dose primaquine for blocking malaria transmission in Cambodia. BMC Med.

[145] Spence PJ, Jarra W, Levy P, Nahrendorf W, Langhorne J (2012). Mosquito transmission of the rodent malaria parasite *Plasmodium chabaudi*. Malar J..

[146] Plouffe DM, Wree M, Du AY, Meister S, Li F, Patra K (2016). High-throughput assay and discovery of small molecules that interrupt malaria transmission. Cell Host Microbe.

[147] Lucantoni L, Silvestrini F, Signore M, Siciliano G, Eldering M, Dechering KJ (2015). A simple and predictive phenotypic High Content Imaging assay for *Plasmodium falciparum* mature gametocytes to identify malaria transmission blocking compounds. Sci Rep..

[148] Delves MJ, Straschil U, Ruecker A, Miguel-Blanco C, Marques S, Baum J (2016). Routine in vitro culture of *P. falciparum* gametocytes to evaluate novel transmission-blocking interventions. Nat Protoc.

[149] Vos MW, Stone WJ, Koolen KM, van Gemert GJ, van Schaijk B, Leroy D (2015). A semi-automated luminescence based standard membrane feeding assay identifies novel small molecules that inhibit transmission of malaria parasites by mosquitoes. Sci Rep..

[150] Almela MJ, Lozano S, Lelievre J, Colmenarejo G, Coteron JM, Rodrigues J (2015). A new set of chemical starting points with *Plasmodium falciparum* transmission-blocking potential for antimalarial drug discovery. PLoS ONE.

[151] Delves MJ, Ruecker A, Straschil U, Lelievre J, Marques S, Lopez-Barragan MJ (2013). Male and female *Plasmodium falciparum* mature gametocytes show different responses to antimalarial drugs. Antimicrob Agents Chemother.

[152] Delves M, Plouffe D, Scheurer C, Meister S, Wittlin S, Winzeler EA (2012). The activities of current antimalarial drugs on the life cycle stages of Plasmodium: a comparative study with human and rodent parasites. PLoS Med..

[153] Makanga M (2014). A review of the effects of artemether–lumefantrine on gametocyte carriage and disease transmission. Malar J..

[154] Kern SE, Tiono AB, Makanga M, Gbadoe AD, Premji Z, Gaye O (2011). Community screening and treatment of asymptomatic carriers of *Plasmodium falciparum* with artemether–lumefantrine to reduce malaria disease burden: a modelling and simulation analysis. Malar J..

[155] Ogutu B, Tiono AB, Makanga M, Premji Z, Gbadoe AD, Ubben D (2010). Treatment of asymptomatic carriers with artemether–lumefantrine: an opportunity to reduce the burden of malaria?. Malar J..

[156] Nunes JK, Woods C, Carter T, Raphael T, Morin MJ, Diallo D (2014). Development of a transmission-blocking malaria vaccine: progress, challenges, and the path forward. Vaccine..

[157] Tiono AB, Ouedraogo A, Ogutu B, Diarra A, Coulibaly S, Gansane A (2013). A controlled, parallel, cluster-randomized trial of community-wide screening and treatment of asymptomatic carriers of *Plasmodium falciparum* in Burkina Faso. Malar J..

[158] Chaccour C, Barrio A, Gil Royo AG, Martinez Urbistondo D, Slater H, Hammann F (2015). Screening for an ivermectin slow-release formulation suitable for malaria vector control. Malar J..

[159] Ouédraogo AL, Bastiaens GJ, Tiono AB, Guelbeogo WM, Kobylinski KC, Ouedraogo A (2015). Efficacy and safety of the mosquitocidal drug ivermectin to prevent malaria transmission after treatment: a double-blind, randomized, clinical trial. Clin Infect Dis.

[160] Tiono AB, Dicko A, Ndububa DA, Agbenyega T, Pitmang S, Awobusuyi J (2009). Chlorproguanil–dapsone–artesunate versus chlorproguanil–dapsone: a randomized, double-blind, phase III trial in African children, adolescents, and adults with uncomplicated *Plasmodium falciparum* malaria. Am J Trop Med Hyg.

[161] Premji Z, Umeh RE, Owusu-Agyei S, Esamai F, Ezedinachi EU, Oguche S (2009). Chlorproguanil–dapsone–artesunate versus artemether–lumefantrine: a randomized, double-blind phase III trial in African children and adolescents with uncomplicated *Plasmodium falciparum* malaria. PLoS ONE.

[162] Jamei M, Marciniak S, Feng K, Barnett A, Tucker G, Rostami-Hodjegan A (2009). The Simcyp population-based ADME simulator. Expert Opin Drug Metab Toxicol..

[163] Fairhurst RM (2015). High antimalarial efficacy of dihydroartemisinin–piperaquine on the China-Myanmar border: the calm before the storm. Am J Trop Med Hyg..

[164] Chavchich M, Van Breda K, Rowcliffe K, Diagana TT, Edstein MD (2015). The spiroindolone KAE609 does not induce dormant ring stages in *Plasmodium falciparum* parasites. Antimicrob Agents Chemother.

[165] Teuscher F, Gatton ML, Chen N, Peters J, Kyle DE, Cheng Q (2010). Artemisinin-induced dormancy in *Plasmodium falciparum*: duration, recovery rates, and implications in treatment failure. J Infect Dis.

[166] Nosten F (2010). Waking the sleeping beauty. J Infect Dis.

[167] Waring MJ, Arrowsmith J, Leach AR, Leeson PD, Mandrell S, Owen RM (2015). An analysis of the attrition of drug candidates from four major pharmaceutical companies. Nat Rev Drug Discov..

[168] Sonoiki E, Palencia A, Guo D, Ahyong V, Dong C, Li X (2016). Anti-malarial benzoxaboroles target *P. falciparum* leucyl-tRNA synthetase. Antimicrob Agents Chemother.

[169] Zhang B, Watts KM, Hodge D, Kemp LM, Hunstad DA, Hicks LM (2011). A second target of the antimalarial and antibacterial agent fosmidomycin revealed by cellular metabolic profiling. Biochemistry.

[170] Heidebrecht RW, Mulrooney C, Austin CP, Barker RH, Beaudoin JA, Cheng KC (2012). Diversity-oriented synthesis yields a novel lead for the treatment of malaria. ACS Med Chem Lett..

[171] Schuurman J, Graus YF, Labrijn AF, Ruuls S, Parren PW (2014). Opening the door to innovation. MAbs.

[172] Blaskovich MA, Zuegg J, Elliott AG, Cooper MA (2015). Helping chemists discover new antibiotics. ACS Infect Dis..

[173] Batool S, Khan ZA, Kamal W, Mushtaq G, Kamal MA (2015). In silico screening for identification of novel anti-malarial inhibitors by molecular docking, pharmacophore modeling and virtual screening. Med Chem.

[174] Kuhen KL, Chatterjee AK, Rottmann M, Gagaring K, Borboa R, Buenviaje J (2014). KAF156 is an antimalarial clinical candidate with potential for use in prophylaxis, treatment and prevention of disease transmission. Antimicrob Agents Chemother.

[175] From Aspiration to Action. What will it take to end malaria? http://www.endmalaria2040.org/. 2016. p. 1–31.

