# Erratum to: New developments in anti-malarial target candidate and product profiles

**DOI:** 10.1186/s12936-017-1809-9

**Published:** 2017-04-18

**Authors:** Jeremy N. Burrows, Stephan Duparc, Winston E. Gutteridge, Rob Hooft van Huijsduijnen, Wiweka Kaszubska, Fiona Macintyre, Sébastien Mazzuri, Jörg J. Möhrle, Timothy N. C. Wells

**Affiliations:** 1 Medicines for Malaria Venture, Route de Pré Bois 20, 1215 Geneva 15, Switzerland; 2 Neglected Infectious Diseases Consulting, Sevenoaks, Kent UK; 3 FSG, Rue de Chantepoulet 25, 1201 Geneva, Switzerland

## Erratum to: Malar J (2017) 16:26 DOI 10.1186/s12936-016-1675-x

After publication of the original article [1], the authors wished to submit a number of minor corrections affecting Fig. 2; Tables 3 and 4. Revised versions of these items are published in this erratum.

**Fig. 2:**
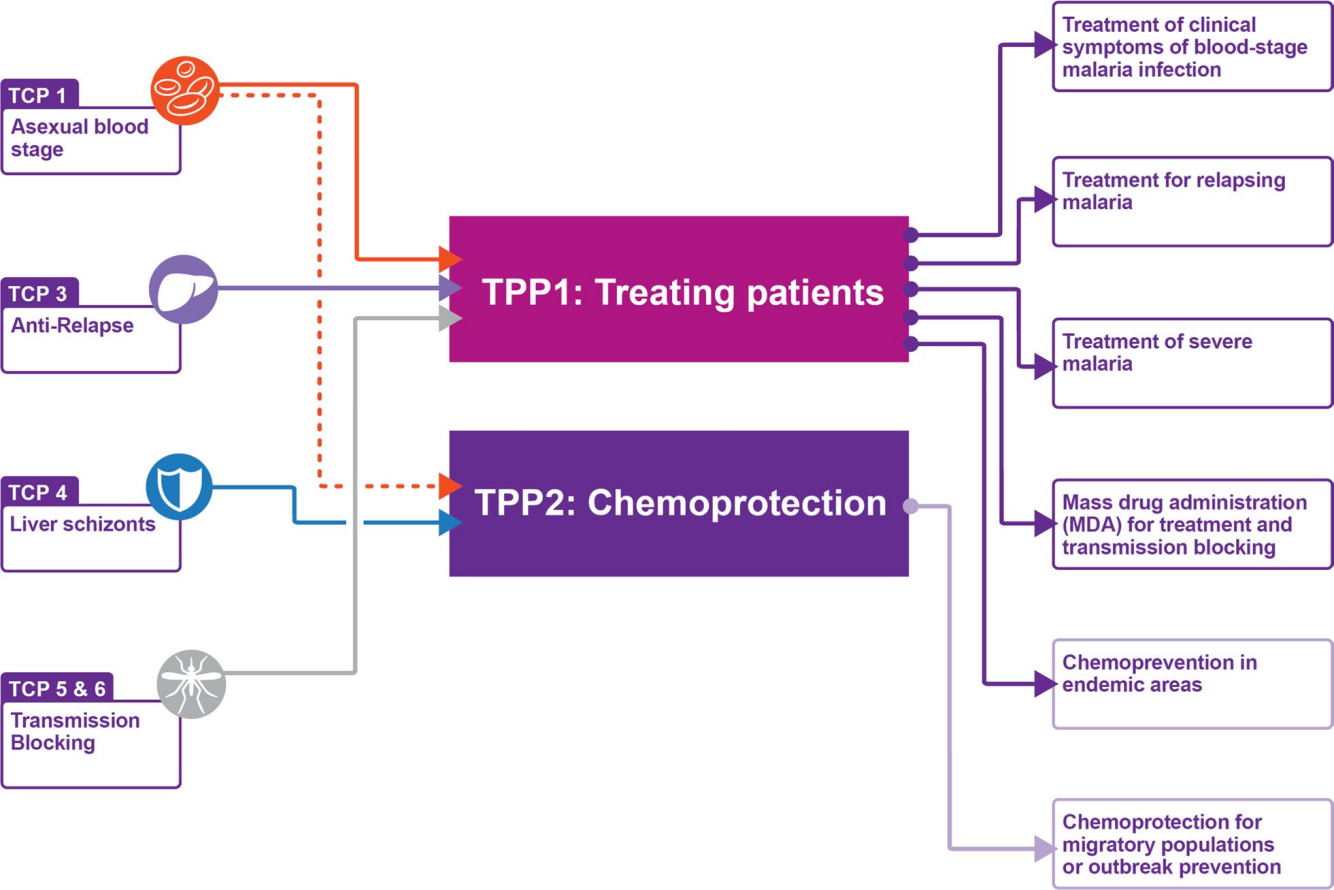
Inter-relationships between the two high-level target product profiles (*center*) with the individual target candidate profiles (*left*) for molecules that are part of the product. The uses for each product are summarized on the *right*

**Table 3:** TPP-2 chemoprotection profiles

Parameter to be demonstrated for the combination in clinical evaluation	Minimum essential	Ideal single exposure chemoprotection
Drug product	For elimination phases at least one of the two compounds also with TCP-4, co-formulated. The other should be a long-lasting blood schizonticide TCP-1	For elimination phases both molecules should have TCP-4 activity, co-formulated
Dosing regimen	Oral, once per week; injectable once per 3 months	Oral once per month; injectable less frequently than once per 3 months
Rate of onset of action	For asexual blood-stage action—slow onset (>48 h)	
Clinical efficacy	≥95% protective efficacy and non-inferior to Standard of Care	≥98% protective efficacy and non-inferior to Standard of Care
Transmission blocking	No	Yes
Bioavailability/food effect	Predicted or measured >30% for each molecule/<threefold	Predicted or measured >50% for each molecule/no significant food effect
Drug–drug interactions	No unmanageable risk in terms of solid state or PK interactions	No risks in terms of solid state or PK interactions
Safety and tolerability	Few and manageable drug-related SAEs in phase III and IV	No drug-related SAEs; minimal drug-related AEs that do not result in study exclusion
Use in patients with reduced G6PD activity	Testing not required; no enhanced risk in mild–moderate G6PD deficiency	No enhanced risk
Pregnancy	Not contra-indicated in second or third trimester	Not contra-indicated in second or third trimester, no suggestion of embryo-fetal toxicity in first trimester in preclinical species
Formulations	Co-formulated tablets or equivalent, with taste-masking for paediatrics if taste is unacceptable to children Long-lasting formulations for intramuscular or intradermal use with low injection volume	Co-formulated tablets for adults. Dispersible or equivalent with taste-masking for paediatrics
Cost of treatment	≤$1.00 for adults, $0.25 for infants under 2 years Similar to vaccine costs for an injectable	Idem
Shelf life of formulated product (ICH guidelines for Zones III/IV; combination only)	≥2 years	≥5 years
Susceptibility to loss of efficacy due to acquired resistance	Very low; no cross resistance with partner	Very low; no cross resistance and orthogonal mechanism from those used in treatment

**Table 4:** TCP-1 profiles, molecules that clear asexual parasitaemia

TCP-1 criteria at human proof of concept	Minimum essential	Ideal
Dosing regimen; adult/paediatric dose	Oral, single dose (predicted) <1000 mg/<250 mg; oral, three doses <400 mg/<100 mg for areas of multidrug resistance	Oral, single dose (predicted); <100 mg/25 mg
Rate of onset of action and clinical parasite reduction ratio from single dose	Rapid clearance of parasites at least as fast as mefloquine (≤72 h from the highest burdens) and projected >10^6^-fold reduction in parasites	Immediate and rapid clearance of parasites at least as fast as artesunate; >projected 10^12^-fold reduction in parasites
Susceptibility to loss of efficacy due to acquired resistance	No fit, transmissible drug-resistant parasites identified in CHMI challenge model; identification of combination partner with no cross resistance	Very low (similar to chloroquine); no cross-resistance with asexual blood-stage combination partner. Resistance markers investigated
Relative clinical efficacy from patients in areas known to be resistant to current first line medications	Clinical efficacy against all known resistance (3-day dosing)	Clinical efficacy against all known resistance (single dose)
Drug–drug interactions	No unsurmountable risks with potential anti-malarial partners	No interactions with other anti-malarial, anti-retroviral or TB medicines
Safety	Therapeutic ratio >tenfold between therapeutic exposure and NOAEL (no adverse effects level) in preclinical studies, and easily ‘monitorable’ adverse event or biomarker for human studies	Therapeutic ratio >50-fold between therapeutic exposure and NOAEL in preclinical studies and easily ‘monitorable’ adverse event or biomarker for human studies
G6PD (glucose-6-phosphate dehydrogenase) deficiency status	Measured—no enhanced haemolysis risk from testing in SCID mice engrafted with human blood from volunteers with reduced G6PD activity; clinical confirmation	Measured—no enhanced haemolysis risk in subjects with reduced G6PD activity, with clinical confirmation
Formulation	Simple and inexpensive to produce, not requiring proprietary methodology or kits; can readily be produced in endemic countries	Simple and inexpensive to produce, not requiring proprietary methodology or kits; can readily be produced in endemic countries
Cost of active ingredient in final medicine	Similar to current medication: ≤$0.5 for adults, $0.1 for infants under 2 years	Similar to older medications: <$0.25 for adults, $0.05 for infants under 2 years
Estimated stability of final product under Zone IVb conditions (30 °C 75% humidity), in final packaging	≥24 months	≥3–5 years
